# Stories in Action

**DOI:** 10.1177/15291006231161337

**Published:** 2023-05-10

**Authors:** James Walsh, Naomi Vaida, Alin Coman, Susan T. Fiske

**Affiliations:** 1 Blavatnik School of Government, University of Oxford; 2 Department of Psychology, Harvard University; 3 Department of Psychology, Princeton University

**Keywords:** stories, narratives, social learning, persuasion, cooperation, media, storytelling, behavioral science

## Abstract

Stories have played a central role in human social and political life for thousands of years. Despite their ubiquity in culture and custom, however, they feature only peripherally in formal government policymaking. Government policy has tended to rely on tools with more predictable responses—incentives, transfers, and prohibitions. We argue that stories can and should feature more centrally in government policymaking. We lay out how stories can make policy more effective, specifying how they complement established policy tools. We provide a working definition of stories’ key characteristics, contrasting them with other forms of communication. We trace the evolution of stories from their ancient origins to their role in mediating the impact of modern technologies on society. We then provide an account of the mechanisms underlying stories’ impacts on their audiences. We conclude by describing three functions of stories—learning, persuasion, and collective action.

For decades, Brazilians have tuned in to watch telenovelas (television soap operas) at 8 p.m. every evening; their appeal cuts across classes. These telenovelas have typically portrayed very small families—which is noteworthy, given that in 1970, as they were becoming popular, the typical woman had almost six children. The overwhelming majority of female characters depicted in telenovelas had no children. Of 115 telenovelas aired by the main television network between 1965 and 1999, 72% featured female characters 50 years old or younger who had no children. Among other female characters, three quarters had only one child (La Ferrara et al., 2012).

One might wonder what effect these depictions of small families could have had on Brazilian society. It certainly correlated with major social change: Between 1970 and 1991, Brazil’s fertility fell by half—from 5.8 to 2.9 children per woman. The drop occurred even though the government made no effort at population control until the late 1970s. To investigate the role of these soap operas, La Ferrara et al. (2012) examined patterns of expansion in television access. In 1970, less than one in 10 Brazilian households owned a television, but by 1991, the figure had increased to more than eight in 10. The network airing the soap operas, Globo, also rapidly expanded throughout Brazil. In 1970, only four areas received a television signal. By 1980, 1,300 areas received a signal, and 3,147 had a signal by 1991. La Ferrara and her colleagues used variation in access to Globo to estimate the effect of exposure to soap operas. Their analysis suggests that these broadcasts resulted in a drop in the fertility rate of approximately 7%. It can be difficult to pin down whether these effects were causal, even with precise covariation. But qualitative traces of soap operas’ effects paint a picture: Approximately a third of the children born in areas with signal access were given names of soap opera characters. In areas with no access, less than a 10th were. The estimated effects were sizeable, almost two thirds as impactful as the effect of the increase in years of schooling during the same period.

What is (and should be) the role of stories in society, and how can a psychological analysis inform this discussion? Stories have generated controversy for thousands of years. For Plato, they misrepresented the truth and failed to inspire virtue or morality. He argued that playwrights and actors should be exiled from Athens. Aristotle famously disagreed. In *Poetics—*his treatise on narrative,^1^ which is still used to teach dramatists today—he proposed that stories were a source of self-understanding. Aristotle maintained that theater, especially tragedy, was necessary to arouse people’s emotions and aid self-understanding (Aristotle, ca. 335 B.C.E./2013; Plato, ca. 375 B.C.E./2000; Stern, 2014). Philosophers and literary theorists have since sought to make sense of the structure and function of stories. But only in the past two centuries have scholars developed the tools to analyze stories systematically (Lévi-Strauss, 1978; Propp, 1968). Even more recently, advances in computational methods and the availability of comprehensive data have transformed scientific understanding of narrative (R. L. Boyd et al., 2020; Michalopoulos & Xue, 2021). Meanwhile, evidence from experimental research has now accumulated to reveal the role of stories in human psychology (Green et al., 2002; László, 2008).

Today’s skeptics echo some of Plato’s complaints. One criticism holds that stories are the antonym of truth. Children are chided for “telling stories”—in other words, lying. More subtly, scholars often imply that stories discourage audiences from rationally assessing systematic evidence by seducing those audiences into the particularities of their narrative worlds. For example, Borgida and Nisbett (1977) found that course recommendations made on the basis of brief personal stories had much larger impacts on students’ course choices than recommendations based on courses’ average scores from evaluations. Stories’ ability to capture their audiences’ attention and emotion mean that even complete fictions can shape how people see the world.

Another critique discounts stories not because they misinform but because they supposedly do not matter. Though this may be hard to believe for scholars committed to the power of narrative, this view pervades subfields of economics, finance, political science, and even history. What really counts, according to this view, are prices, technologies, and the allocation of material resources. In this view, people are rational actors, unpersuaded by rhetoric or advertisements. They rigorously extract only the data they need—whether the source is a story, a recipe, an argument, or a formula—to accurately update their beliefs and pursue their interests (Stigler & Becker, 1977).

This article refutes these two arguments. First, stories do matter. This review shows that stories have played a key role in the development of modern society and specifies how they can improve government policy. Moreover, psychological science is beginning to understand more precisely *why* they matter. Second, far from being vehicles of mistruth, stories are in fact a vital communication tool that people use to pragmatically solve a host of social and developmental problems—from teaching children to read to coordinating large-scale social activities. We nevertheless concede that stories—their nature and function—are poorly understood by government policymakers. To this end, this review brings together research from psychology, behavioral economics, and related fields to lay out how stories can be harnessed more effectively to improve government policy design.

## Overview

After this introductory section, the second section considers the implications of the science of stories for policy design. Narratives can improve the effectiveness of two standard policy instruments: incentive and information provision. Stories can make incentives more effective by communicating the meaning that motivates them. Stories can improve information campaigns by communicating easily digestible, generalizable information to large audiences and by addressing sensitive issues.

The third section defines stories and describes their key features. Stories are concrete—they describe specific events. Stories depict agency—they are about protagonists and their goals. Stories contain causal sequences—they provide a template for how action unfolds. Stories have their own logic, and people evaluate them differently from other information structures.

The fourth section traces the evolution of stories. It reviews the origins of storytelling and how advances in writing systems transformed humans’ capacity to communicate sophisticated stories at scale. It then discusses how stories were democratized with the invention of technologies such as the printing press, radio, television, and social media. As the reach of stories expanded, their impacts on social and political life have become increasingly visible.

The fifth section describes the story mechanisms that impact cognition: engagement, identification, and meaning construction. Stories engage, or equivalently transport, when they captivate attention and emotionally absorb people, for example, by creating suspense. Stories also invite people to identify with their characters. In doing so, the audience learns from the perspectives of the protagonist. Finally, stories embed causal models that offer people ways to make sense of the world. These three mechanisms are key paths via which stories lead people to update their beliefs, attitudes, and behaviors in story-consistent ways.

The sixth section zooms out to describe three societal functions of stories: social learning, deliberate persuasion, and collective action. First, it discusses how stories aid social learning and teaching. Stories enable social learning without direct observation and facilitate teaching by making information more memorable and understandable. Second, narrators use stories to affect attitudes, beliefs, and behavior. Stories are persuasive because they reduce reactance, they convey causal models, and they facilitate vicarious engagement. Third, stories address collective action problems, such as coordination challenges and social dilemmas. Stories do so by establishing common knowledge: shared expectations, explanations, reputations, and identities.

## Stories and Public Policy

Societies have used stories as vehicles for communicating important information for thousands of years. Although they are commonplace in politics, stories feature only peripherally in government policymaking. Policymakers are trained to design laws and social programs on the basis of the principles of economic theory. In this paradigm, people are assumed to be economically rational actors. They maximize their expected utility by calculating the costs and benefits of different courses of action. They also have the capacity to process unlimited information, enabling them to formulate beliefs as Bayesian statisticians (Stigler & Becker, 1977). This approach assumes not only that people are self-interested but also that they think in what Jerome Bruner calls the “logico-scientific” mode—thinking through arguments in terms of their logical implications and evaluating the strength of evidence substantiating different claims (Bruner, 1986). The model provides governments with two main policy tools: incentives and information. In recent years, behavioral policymakers—scientists and practitioners housed in government units focused on behavioral change—have incorporated psychological insights into policy on the basis of the idea that people are biased toward heuristics and shortcuts. For example, sending timely reminders improves adherence even to lifesaving drugs, and making pension contributions the default increases savings, even though having to opt in or opt out should not affect rational decision-making on such a significant issue. This article builds on dual-process frameworks in behavioral policy to emphasize that people think narratively. This has several implications for policy.

### Incentives

Stories can make incentives, the cornerstone of modern government policy, more effective. Policymakers use incentives to shift behavior by taxing or subsidizing activities (e.g., by putting a levy on alcohol or offering people lower interest rates for educational loans). They also use them when threatening to impose fines or jail time for people found in violation of regulations or laws. According to economic theory, laws discourage criminal behavior by making it costly. People are assumed to weigh up the costs by combining the likelihood of being punished with the magnitude of the punishment (Becker, 1968). Stories can make incentives more effective in several ways.

First, stories can establish the meaning of incentives. Rational actors, or homo economicus, make decisions by coolly calculating their costs and benefits. Human beings, on the other hand, are cultural beings. They make decisions, even about how to respond to prices, by applying meaning to the context (E. Anderson, 1995; Sunstein, 1994). When people misconstrue the meaning of an incentive, it can backfire. A study from Israel examining the effect of day-care fines for parents who pick up their children late is illustrative. When randomly selected day cares introduced a fine to discourage parents from arriving late, parents arrived even later than they did at day cares in the control group. The parents, the authors argue, interpreted the fine as a price. Parents did not feel comfortable taking advantage of teachers’ patience, but once the fine was introduced, they felt more comfortable paying to arrive late (Gneezy & Rustichini, 2000). Stories can be effective ways of communicating the rationale for fines, taxes, and subsidies. Consider, for example, fines for speeding. People may “price in” the cost of occasional tickets for speeding—determining that the occasional ticket is worth time saved. However, if speeding tickets are accompanied by campaigns containing stories about the rationale for speeding tickets (i.e., to discourage speeding because it can lead to fatal car accidents), the social meaning of being fined may lead people to avoid the fee because of the moral weight associated with it.

Second, stories can make incentives more effective by reifying the implications of the cost. One prediction of the economic approach to crime and punishment is that longer sentences should discourage criminal behavior. Empirical evidence for this prediction is weak, however (Chalfin & McCrary, 2017). Moreover, incarceration is expensive to the state and to the incarcerated individual. One explanation for why longer or more punitive sentences are weak deterrents is that although people generally know what kinds of activities are illegal, they are not good at assessing the probability and severity of punishment. They tend to qualitatively base their estimates on actual or vicarious experiences (Apel, 2013). This presents an important entry point for stories, which can shape how people perceive both the likelihood and cost of punishment. For example, stories could convey the life events missed through incarceration. A mere statement that being convicted of a particular law results in a 10- to 15-year sentence does little to bring to life the experienced cost of prison. But stories can do more than this. They can also zoom into contexts in which people have to decide whether to engage in illegal behavior and model how to escape situations in which there are pressures toward criminal behavior.

### Information provision

Governments can use stories to improve how they communicate information, another key function of government. Governments are responsible for informing citizens about the safety of consumer products and for advising people how to access social services, such as education and training opportunities. They give people instructions on how to vote and provide guidance on how to stay safe from disease. The norm is to focus on facts, statistics, and instructions—aiming to help people make decisions based on good evidence derived from systematic data collection. Stories are rarely representative accounts of the real world that depict the average person undertaking the most common activities. They are often fictional—explicit mental constructions of things that never actually happened. Government policymakers should be careful, of course, to ensure that the information they convey is based on good evidence. Stories can nevertheless be useful for several reasons.

First, people are adept at recovering generalizable information from stories that they can then adapt and use in their day-to-day lives. Government communication often aims to convey complex information about how to undertake activities (e.g., registering a company, paying taxes, or applying to college). When these processes are presented as abstract lists of generic rules, requirements, steps, or principles, they may be so cognitively taxing that they discourage people from even considering undertaking the action. The process can, alternatively, be described in narrative terms. Consider, for example, the story of Hannah, who wants to start a dog-walking business but has no experience registering a company. She looks up the form online and at first is daunted by the amount of detail requested but then quickly realizes it is manageable. She enters her details and goes to the bank to acquire the required documentation, then submits the application. Several weeks later, she is the owner of a registered company. Whether the story is literally true or not has no bearing on the audience’s ability to register a company. Rather, the story’s effectiveness will be determined by its ability to engage the audience and convey key causal relationships in a lifelike way.

Second, people are attracted to stories as to virtually no other information source. This makes them distinctively scalable ways of reaching large numbers of people. High-quality stories often reach remarkably large audiences, so the distributed cost per person is very low. BBC Media Action, a nonprofit focused on using stories to promote social development, reaches approximately 100 million people every year around the world, a number that resembles the population size of large countries. Stories’ reach means that they not only are an efficient way of communicating information to the public but also can be distinctively effective at solving collective action problems, in which everybody needs to coordinate on a shared understanding. Stories can coordinate groups around national efforts toward, for example, environmental protection, national defense, or public health.

Third, stories can depict a multitude of possibilities, making them an effective route to counteract unequal social structures. A key policy goal for most democratic governments is to make society fairer and to promote equality of opportunity. Social rigidities are one major obstacle to this goal. For example, children who grow up in families and communities where pursuing higher education is normal can imagine without difficulty what life would be like at college. They learn through relationships and networks how to prepare their applications. When they arrive, they know what courses to take, and when they finish, they know the kinds of jobs to seek. The problem is not that advantaged and disadvantaged groups misperceive the world. Rather, the problem is that the world is so segregated that it limits people’s access to possible worlds. In the real world, families in disadvantaged neighborhoods generally do not know many people who went to college, and college-bound families rarely spend time in areas of socioeconomic deprivation. In these cases, it is precisely because the credibility of fictional stories is not based on their literal truth that their representations can emancipate people.

Fourth, stories are an adaptable tool for navigating sensitive cultural issues in which governments face crises of trust—such as election integrity, police conduct, and the safety of vaccines. Stories can do this in several ways. One way, for example, is that stories can reduce reactance. When information is presented to people in the form of a story, they are less likely to feel that they are being manipulated and to counterargue (Kreuter et al., 2010). Another way is that stories can signal to diverse audiences—demographic or political—that the communicator understands their perspective. This can be done, for example, by telling a story from the point of view of a member of a particular group, or it can represent a protagonist having a moment of realization about a particular group’s truth. This can be especially powerful when groups feel that their perspective on an issue is being misrepresented or caricatured by the media or decision-makers.

In summary, stories can be an important addition to the policymaker’s toolbox. They can make incentives more effective by communicating their meaning and by reifying the implications of incentives. They can make information campaigns more effective, despite the fact that they are not systematic representations of the truth. This is because (a) people are good at pragmatically extracting useful information from stories, (b) people are more drawn to stories than other forms of information, (c) stories can help people imagine a reality beyond the status quo, and (d) stories enable communicators to establish trust with their audiences.

## The Definition and Structure of Stories

Stories are information structures, but so are all mental representations. A central challenge in empirical work on stories is distinguishing narrative from other forms of communication, such as instructions, arguments, and statistical tables. Cognitive psychologists have studied how stories are represented in the mind since the 1970s and 1980s (Black & Wilensky, 1979; Mandler, 1982; Mandler & Johnson, 1977; Rumelhart, 1975; Stein, 1982; Stein & Trabasso, 1981; Trabasso & van den Broek, 1985). Dahlstrom (2014) emphasizes three key features of stories: (a) Stories depict temporal events, (b) stories are concerned with goal-directed agents, and (c) stories represent causally related sequences.

Although these features are guideposts, defining stories in terms of necessary and sufficient conditions can lead to counterintuitive conclusions. On the one hand, some communication may meet these criteria but not be recognizable as a story—for example, simple descriptions of human behavior. On the other hand, communication may lack these features—for example, visual art—but implicitly contain stories. For this reason, narrativity may be best thought of as a continuum based on family resemblances (Stein, 1982; see also Rosch & Mervis, 1975). Prototypically, stories represent the vicissitudes of human action—either implicitly or explicitly referring to causal sequences of events and agents undertaking goal-directed behavior (Mandler & Johnson, 1977; Prince, 1973; Stein, 1982).^2^

To illustrate how vicissitudes (i.e., sudden changes in circumstances) increase the prototypicality of stories, consider a simple passage: “John was out of milk, so he went to the store to pick some up.” The passage meets the basic criteria for a narrative. There is a temporal event (buying milk at the store), there is a protagonist (John), and there is a causal sequence of events (John went to buy milk *because* he was out of it). But the passage only barely resembles a story. A reader is unlikely to be transported into the story world, to identify with John, or to derive meaning from the information. Imagine the passage continued with,

As he deliberated over whether to go for the oat or almond variety, he noticed something was amiss. The cashier had a look of terror on her face. Suddenly, John realized he was in the middle of an armed robbery. As he dropped to the floor, distant sirens began to get louder.

The inciting event makes the passage more prototypically storylike. We are transported into the scene and wonder what will happen next. Depending on what John decides to do, we might extract some useful lesson.

Another consideration is that stories often reference implicit knowledge: Audiences must draw on background information from their own memory to construct meaning out of the chain of events. The passage “the cashier had a look of terror on her face” implies that something frightening has happened because frightening events terrify people. The passage “distant sirens began to get louder” implies that the police were on their way. Similarly, the audience may reference other stories in making sense of the plot. For example, if the robbers were zombies, the audience might struggle to understand why John would protect himself with garlic (alleged to repel vampires). One challenge is that stories often mean different things to different people, depending on the references they use to interpret the story. To judge the plausibility of stories, people draw on their repertoire of cultural knowledge, which varies from group to group (Polletta, 1998). In Currie’s (1990) account, readers relate both to the text and to their construction of the author’s intent. This particularly matters in regard to stories used in the public interest. For example, John’s race or the identity of the author would inform audience interpretation of whether John is equally afraid of the police and the robbers.

### Events

The most basic feature of stories is that things happen. They are concerned with particular or concrete representations, set in a time and place. The story of Rapunzel is about a lonely princess locked away in a remote tower tucked into the woods. The textual scene “a long time ago in a galaxy far, far away” transports the mind into the fantastical world of *Star Wars*. “The convenience store exterior was covered with local gang graffiti” conveys a run-down urban settings. Stories’ concreteness contrasts with abstract representations, such as equations, theorems, proofs, and many arguments, which make no reference to time or space. Stories work in precisely the opposite way. They draw audiences into the specifics of the scene. Bruner (1986) writes that narratives “strive to put timeless miracles into the particulars of experience, and to locate that experience in time and space,” whereas logical or scientific modes of thought conversely aim “to transcend the particular” (p. 13).

### Agents

Agency is a second distinguishing feature of stories. Stories deal with protagonists’ desires, beliefs, and actions. The protagonist may be a person, a group, an animal, some fictional species, or even an inanimate object that has been anthropomorphized.^3^ The philosopher Daniel Dennett calls this “the intentional stance.”^4^ As a result of their depiction of agency, stories engage with subjectivity in a way that nonnarrative forms do not. Stories invite their audience to see things from the protagonist’s point of view. Actors’ goals and desires, and what they think, feel, know, and (sometimes crucially) do not know, are often central to narrative (Bruner, 1986). One advantage of dealing in subjectivity is that stories can represent multiple perspectives, conveying different actors’ goals, desires, and beliefs—without the need for them to be complete.

### Causal sequences

Causal sequences are the third feature of stories. Narratives give coherence to the organization of events (Black & Bern, 1981; Trabasso & Sperry, 1985; White, 1990)—a logic to their unfolding. Decisions are made, plans are disrupted, hearts are broken, and plots are foiled. Although people assess the strength of regular arguments by referencing classical standards of consistency or empirical proof, they assess stories on the basis of their lifelikeness (Bruner, 1986) or at least their plausibility within the narrative world. Actions have consequences. Betrayal triggers revenge. Bravery elicits admiration. Stories must resemble human life to be understood.

Plots engage their audience by creating and resolving uncertainty. Just as audiences quickly zone out if stories are too absurd (the robbers are not only vampires but also unicorns), audiences are also put off by mundane descriptions of everyday life (e.g., John goes to the store, buys milk, and goes home; Nyhof & Barrett, 2001). If we learn that the robbers kidnap the cashier, we want to know the outcome. The uncertainty draws us in. Yet the range of ways in which we are willing to engage with this uncertainty is limited. In line with this, literary scholars had long argued that stories follow particular formulas.

### The structure of stories

**Fig. 1.**
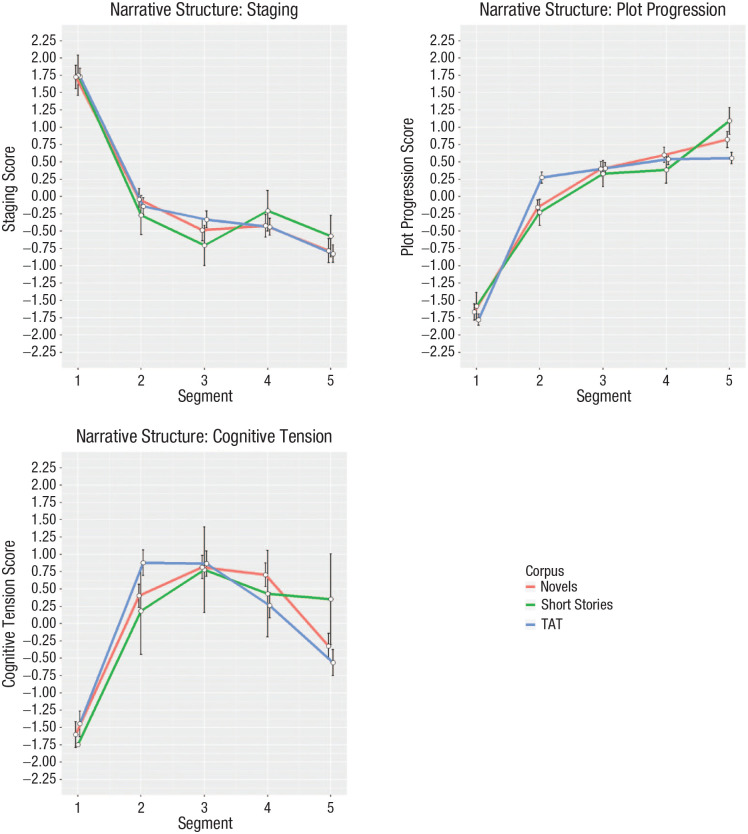
The structure of stories. Results from text analyses of three aspects of narrative structure (staging, plot progression, and cognitive tension) are shown separately for three corpora of texts. The red lines reference analysis on novels. The green lines reference analysis on short stories. The blue lines reference stories written by Internet users in response to thematic prompts (thematic appreciation test [TAT]). The figure shows that, common across all corpora, language used to construct situations occurs most frequently at the beginning of the story, language used to establish the momentum of the plot increases quickly then slowly as the story develops, and language describing cognitive tension rises and then falls. Error bars show standard errors. (Source: R. L. Boyd et al., 2020.)

Many scholars have sought to identify an underlying structure of good stories. Recently, a computational project examined a corpus containing millions of texts shed light on this (R. L. Boyd et al., 2020). The investigators linked word types to different components of narrative. Recall that stories are concrete, set in a time and place. The analysis shows that stories generally establish concreteness early on in the text as they stage events and scenes—laying out the backdrop and establishing locations, characters, and their relationships to one another. Articles and prepositions feature heavily in these early parts. The second feature of narratives is agency. The investigators explored this by looking at the degree of cognitive tension in the text. They found that stories start off with low levels of cognitive tension, but they build and climax in the middle. The final feature is that stories contain causal sequences of events. One might think of this as the plot. The investigators showed that the plot develops progressively through the text (using pronouns and auxiliary verbs) and climaxes at the end. This suggests that, as literary scholars have long argued, narratives follow a basic structure (Fig. 1).

In summary, the distinctive feature of stories, in contrast to other information structures, is that they portray events, they contain agents, and they are organized via causal sequences. Stories have a number of other important attributes, however. One is that audiences generally must draw on detailed implicit knowledge to make sense of stories. Another is that stories can convey multiple subjective perspectives, whereas other forms of communication often present information in an objective manner. Finally, audiences process the coherence of stories against an internal narrative logic; this stands in contrast to forms of communication that are evaluated against classically logical or empirical standards.

## The Evolution of Stories

As best investigators can tell, storytelling is a universal human practice (D. E. Brown, 2017; Hogan, 2003). Most daily conversation consists of narratives of some form or other (Dunbar et al., 1997). People are routinely motivated to gossip about others (Foster, 2004) and enjoy sharing their own experiences (Tamir & Mitchell, 2012). A large body of psychology has explored how people use narratives to construct the self (McAdams, 1993, 2001). The practice of narrativizing identity enables people to organize their past, to imagine possible futures, and to give meaning, purpose, and unity to life (McAdams & McLean, 2013; Singer, 2004). Indeed, establishing coherent personal narratives forms the basis of some mental health therapies (Pennebaker & Seagal, 1999). For decades, many psychologists have argued that much if not most of human thinking takes place in narrative form (Bruner, 1986; S. G. B. Johnson et al., 2022; Mandler, 1984; Sarbin, 1986; Schank & Abelson, 1995). Some have argued that all thinking takes place narratively, though this is surely wrong (W. F. Brewer, 2014). For example, representations of shapes, logical relations, and physical laws need not be narratives.

People are drawn to storytellers. The cultlike status of celebrities (Brooks, 2021; McCutcheon et al., 2002) may rest on virtually all of them being in the business of storytelling. The polling company YouGov tracks and ranks the fame and popularity of notable people in the United States. As of 2022, almost all of the 10 most famous people in the United States today had either written popular memoirs or performed in movies or reality TV shows prior to achieving major notoriety. All of the 10 most popular people in the United States were also actors, though one was primarily a musician.^5^ The influence of celebrities on attitudes, beliefs, and behavior is so strong that their endorsements are a key part of businesses’ marketing strategies (Erdogan, 1999; Knoll & Matthes, 2017).

Although one might think of the cult of celebrity as a quintessentially modern phenomenon, the attraction to storytellers may be rooted in ancient ways of life. One way to investigate this is to look at communities living today as most people did thousands of years ago. A recent study of the Agta, a Filipino hunter-gatherer population, assessed the role of storytelling in their communal organization (D. Smith et al., 2017). The authors found that stories played a key role in regulating norms and conveying information to group members, especially to children. Moreover, when members of the population from 18 different camps were asked who they would most like to live with, skilled storytellers were almost twice as likely to be chosen than nonskilled storytellers. Storytelling ability was more predictive than skill in hunting, medicinal knowledge, and camp influence. This relationship held even after the authors controlled for factors such as kinship, age, and sex. The preferences that group members stated were backed up by consequential outcomes, too: Good storytellers had significantly more children.

**Fig. 2.**
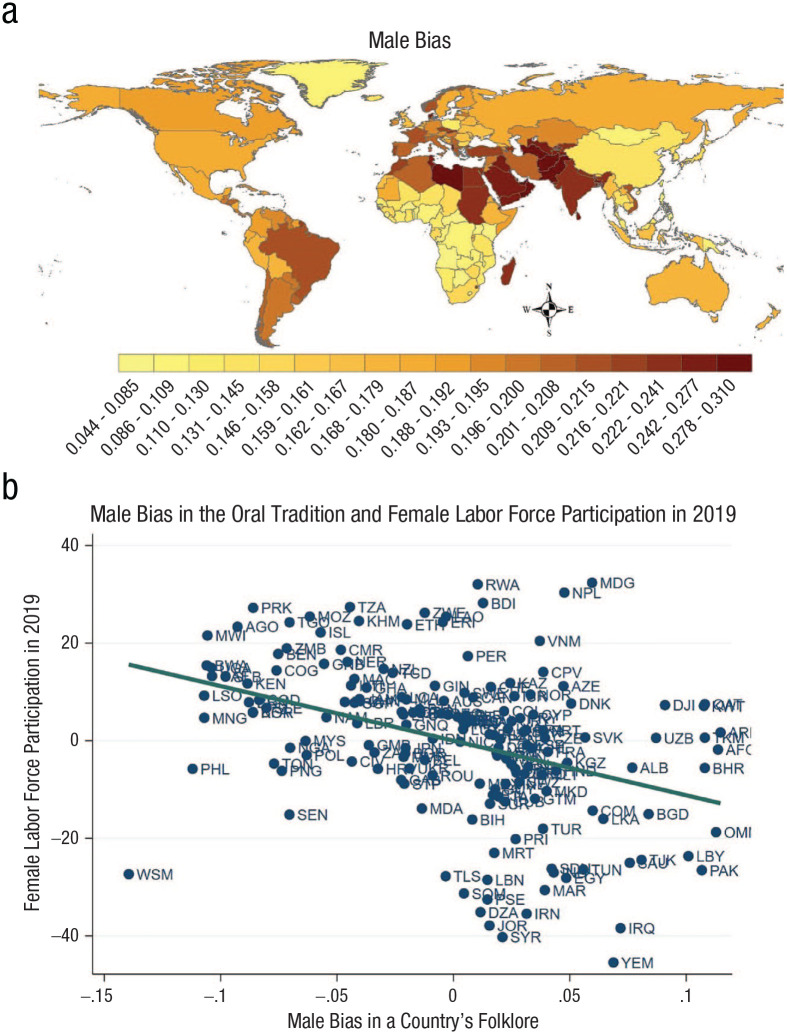
Gender bias in folklore and female labor force participation. The map (a) shows the cross-country rates at which men, relative to women, are depicted as more dominant and physically active and less submissive and domestic. The scatterplot (b) shows residuals from a regression testing the relationship between male bias in each country’s folklore and female labor force participation in 2019. The line indicates the ordinary least squares regression, conditional on continental fixed effects, log year of earliest publication, and log number of publications. For an explanation of the country abbreviations, see the source (Michalopoulos & Xue, 2021).

One reason that storytellers may be so valued is that stories are a key vector for maintaining culture through generations. This is consistent with recent work showing the relationship between countries’ folkloric traditions and contemporary moral values. Michalopoulos and Xue (2021) examined a catalog of folklore developed by the Russian folklorist Yuri Berezkin that contains more than 2,500 motifs from 958 groups around the world.^6^ Using the presence of gendered stereotypes as their independent measure, the authors analyzed whether gender portrayals in the motifs predicted contemporary attitudes toward women. They coded male stereotypicality on the basis of depictions of men as dominant, physically active, aggressive, and arrogant and coded female stereotypicality on the basis of depictions of women as domestic, emotional, beautiful, dependent, and submissive.^7^ They found that women are systematically less integrated into the labor market in societies with more gender bias in their folklore (i.e., they feature more images of dominant and physical men and domestic women). To give an example, the Philippines has negligible bias against women in its traditional folklore, whereas measures of gender bias in Afghanistan are twice that of the average country (Fig. 2).

### Origins of storytelling

Many researchers have set out to explain how humans came to be so enthralled with stories, not least because being lost in imagination may seem to be a maladaptive strategy for a species facing immediate survival pressures and risks (B. Boyd, 2009; Carroll, 2004; Dissanayake, 1988; Gottschall et al., 2005).^8^ Brian Boyd summarizes a potential route on the journey to storytelling:

The pressure to pool ever more information, even beyond currently shared experience, led to the invention of language. Language in turn swiftly unlocked efficient forms of narrative, allowing early humans to learn much more about their kind than they could experience at first hand, so that they could cooperate and compete better through understanding one another more fully. . . . Once the strong existing predisposition to play combined with existing capacities for event comprehension, memory, imagination, language, and narrative, we could begin to invent fiction, and to explore the full range of human possibilities in concentrated, engaging, memorable forms. First language, then narrative, then fiction, created niches that altered selection pressures, and made us ever more deeply dependent on knowing more about our kind and our risks and opportunities than we could discover through direct experience. (B. Boyd, 2018, abstract)

According to one recent hypothesis, elaborate forms of storytelling emerged through conversations around the campfire (Dunbar, 2014). Evidence for the association between fire and storytelling comes from the work of anthropologist Polly Wiessner, who spent several decades living with the Ju/’hoansi Bushmen, a forager society of Botswana and Namibia. Over approximately 40 years, Wiessner collected data on the Bushmen’s conversations. She found that during the day, the Ju/’hoansi focused on economic issues, jokes, and gossip aimed at regulating behavior. These types of communication contain basic narrative elements. At night, however, more than 80% of conversations focused on elaborate stories: hunting ventures, meat fights, murders, marriages, bushfires, getting lost, and births (Wiessner, 2014).

**Fig. 3.**
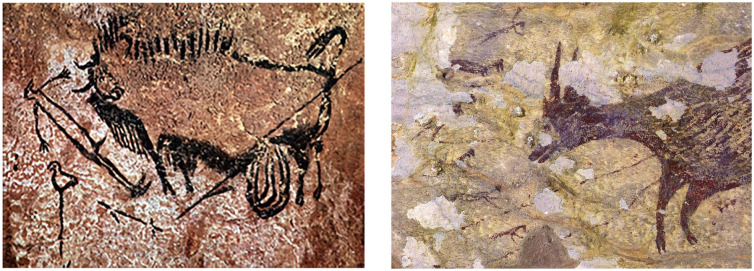
Evidence of ancient storytelling: the oldest known hunting scene from Europe (~17,000 years old; left) and a much older hunting scene from Indonesia (> 40,000 years old; right). (Sources: Wikimedia, 2022, and Ratno Sardi, ©2019, respectively.)

Unfortunately, the campfire thesis cannot date the origins of storytelling. Humans have had the ability to control fire for 1 to 2 million years (Berna et al., 2012), whereas the capacity for language is estimated to be only about 100,000 years old (Berwick et al., 2013). Ancient cave paintings allow for much more precise lower-bound estimates. Cave art is thousands of years old and has been found on every continent. To put their age in perspective, consider that some cave paintings depict extinct animals such as the woolly mammoth (Gross, 2020). Although much of the cave art that has been discovered is indicative of complex creative thought, only a small fraction contains hallmarks of narrative (i.e., representations of scenes or events). Perhaps the most widely known example of narrative cave art comes from artwork found in the 1940s in Lascoux, France. This includes a 17,000-year-old scene (Fig. 3, left) that seems to depict a wounded bison charging down a bird-headed shaman (Davenport & Jochim, 1988). More recently, however, evidence of much older narrative art has been found in cave art in Indonesia (Aubert et al., 2019). This art, estimated to be more than 40,000 years old, displays a scene with tiny figures armed with spears and ropes who appear to be hunting a wild cow (Fig. 3, right).

### Writing systems and their implications

We can only speculate about the narrative content of these images. The advent of writing systems approximately 5,000 years ago radically improved the effectiveness of story transmission. The earliest written story of note is the *Epic of Gilgamesh* (1999), a mythic poem written on 12 clay tablets. The standard form was compiled by a Mesopotamian priest around 1200 B.C.E., but its origins are hundreds of years older.^9^ The *Epic* focuses on the adventures of Gilgamesh, a Sumerian king who is two thirds god, one third human. The story opens with Gilgamesh as an unworthy king. He disrespects the gods, his subjects are unhappy, and he delights in jus primae noctis—“the right of first night”—a rule that entitles him to rape newlywed brides. In a desire to achieve fame and renown, Gilgamesh and his friend Enkidu embark on a journey to the Cedar Forests and defeat the forest’s guardian Humbaba, a monstrous giant. Although they are successful, Gilgamesh’s friend Enkidu is eventually killed by the gods, propelling Gilgamesh into grief and motivating him to seek out a path to everlasting life. When he ultimately fails, he comes to terms with his human mortality and, in doing so, finally finds true wisdom. The story shows that humans have been engaged in sophisticated narrative thought for thousands of years, grappling with important psychological concerns: the drive to attain power, the importance of friendship, and the tragedy of loss, as well as the inevitability of death (Abusch, 2001).

As writing systems evolved, the sophistication of storytelling advanced, too (Puchner, 2018). For thousands of years, writing consisted of symbols that stood for particular things in the world. For example, writing used in Mycenaean Greece up to the 12th century B.C.E. contained symbols for ox, jug, and barley. Around the 8th century B.C.E., the Phoenicians switched from linking symbols to meaning toward a system that connected symbols with sounds. This reduced the number of required signs from hundreds (or sometimes thousands) to a few dozen. Although the Phoenicians included only consonants, the Greeks then improved on the Phoenician system by adding vowels. Not long after, the Greeks began to use their new alphabet to document the stories of the Trojan war.^10^ This period saw the production of many of the world’s most important texts. It is in Greek that the Homeric epics, the *Iliad* and *Odyssey*, were codified in text, that Plato recorded the arguments of Socrates, and that the New Testament described the life of Jesus Christ. The stories contained in these texts set the cultural foundation for Western life for the two millennia that followed.

### The rise of the printing press

**Fig. 4.**
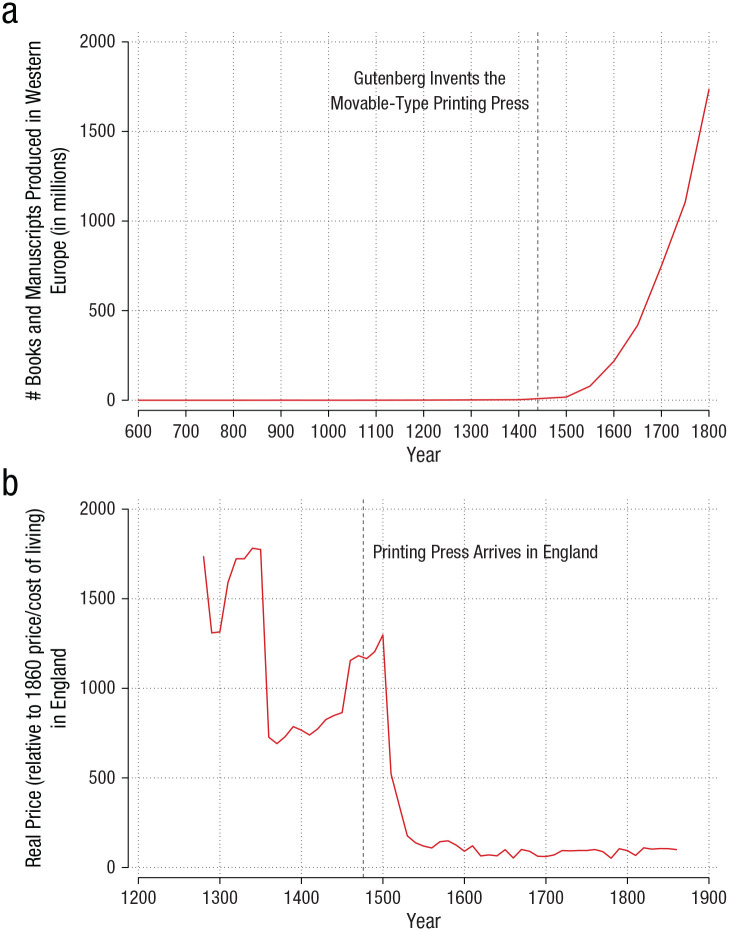
The mass production of books. Graph (a) shows estimates of the real price of books in England from the 1200s through the 1800s. The price fell dramatically between 1350 and 1550. Scholars speculate that the first decline, around 1350, was driven by a transition from parchment to paper—though data from earlier periods is less reliable. Graph (b) shows the number of books and manuscripts produced in the 100 years preceding each time point on the *x*-axis. Book production exploded in the middle of the 16th century. (Sources: AI Impacts, 2020; Buringh & Van Zanden, 2009; Clark, 2004.)

The power of stories accelerated yet again when new technologies enabled the use of writing for mass communication. In 1440, German inventor Johannes Gutenberg developed a printing press capable of mass production. Though the press had already been invented in China, its alphabet contained thousands of symbols, which restricted its applications. As Figure 4b shows, the price of books collapsed after the advent of the printing press, which was quickly followed by an enormous increase in book production (Fig. 4a). This period also saw storytelling flourish, as William Shakespeare produced plays that delved into the frailty of the human condition and Miguel de Cervantes wrote the first modern novel, *Don Quixote*. These works continue to be rated by many as the highest accomplishments of theater and literature. In 2010, when Google embarked on a mission to scan books at mass scale, they calculated that approximately 130 million books had been published since Gutenberg’s invention of the printing press (Taycher, 2010).

**Fig. 5.**
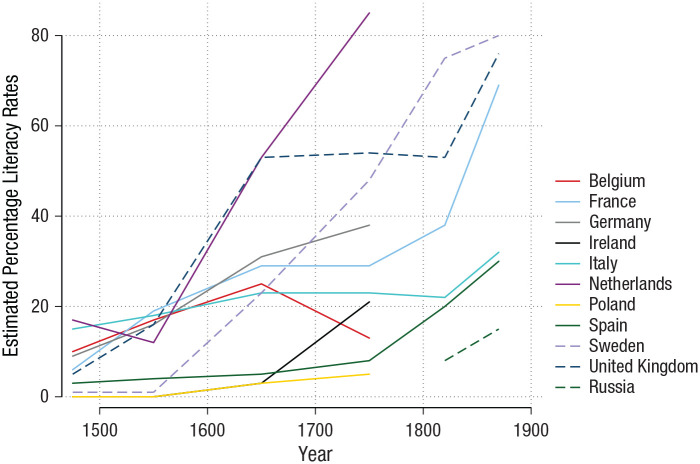
Literacy rates in Europe. The estimated levels of literacy in different European countries are shown for each 50-year period between 1550 and 1900. Results are based on book-publishing data and measures of literacy. (Sources: Buringh & Van Zanden, 2009; Roser & Ortiz-Ospina, 2018.)

Perhaps more important, the printing press had a profound effect on literacy. Before its invention, rates of literacy in societies are thought to have been never more than about 10%. In the 16th and 17th centuries, rates of literacy began to explode in Europe in response to the Protestant Reformation, which promulgated the doctrine that individuals should develop a personal relationship with God and Jesus. Out of this need came the principle of *sola scriptura*—by scripture alone—which posits the Bible as the only infallible source of authority for the Christian faith. This had the effect of encouraging Christians to learn to read. By 1445, Gutenberg had published the first mass-produced Bible, and by the end of the century, presses were operating throughout Western Europe. Laypeople began to read the Bible, and the stories within it, themselves. By 1750, it is estimated that almost 90% of adults living in The Netherlands were literate (see Fig. 5; Henrich, 2020).

The printing press also enabled pamphlets and newspapers to circulate regularly. Newly established coffeehouses throughout Europe were an early venue for their distribution. In English coffeehouses, “news could be consumed in a variety of different forms: in print, both licensed and unlicensed; in manuscript; and aloud, as gossip, hearsay, and word of mouth” (Cowan, 2008, p. 87). Some social historians, influenced by the social theorist Jurgen Habermas (1991), hypothesize that these developments were key to the emergence of a public sphere in Western society (see also Pincus, 1995). By the 19th century, new processes for making paper from pulp, the advent of the telegraph, and reduced postage costs led to a rapid expansion of newspapers in the United States. Newspapers quickly became a battleground for partisanship, and many papers were bully pulpits for political leaders. In 1870, roughly one in 10 newspapers were independent. Toward the end of the 19th century, newspapers slowly became more independent, and by 1920, independence had become the norm (Gentzkow et al., 2006). Newspaper readership also rose throughout this period, and by 1920, the average urban adult in America was purchasing more than a newspaper a day (Chandra & Kaiser, 2015).

Mass print’s influence extended beyond Europe and the United States. In sub-Saharan Africa, access to printing was heavily shaped by colonialism. Native Africans’ access to printing was restricted to sources made available by Protestant missionaries who brought presses with them to print educational material and Bibles. Throughout the 19th century, missionaries acquired printing presses and established schools to train local people in printing. The overwhelming aim was to promote their religion, but proximity to printing nevertheless had significant implications for Africans’ access to newspapers. The first newspaper intended for Black readers was published in 1837, the first African newspaper edited by Africans appeared in 1876, and the first Black-owned newspaper in South Africa, *Imvo Zabantsundu* (*African Opinion*), was published 8 years later in 1884. All of these events occurred in regions close to missions. No newspapers were published in regions without Protestant missions until the early 20th century, and no Indigenous-run newspapers were created until after the first World War. Contemporary data from the Afrobarometer show that these patterns had long-lasting impacts. Africans who today live close to the location of a mission with a printing press are significantly more likely to read the news, to have higher trust in others, and to have higher education. In democracies, Africans close to missions that had printing presses are more likely to participate politically. These effects occur only for missions with printing presses; proximity to missions without presses, whether Catholic or Protestant, has no impact on contemporary newspaper readership (Cagé & Rueda, 2016).

### Broadcasting stories

The dominance of newspapers in the United States began to decline in the early 20th century. Radio technologies, originally developed for military and maritime purposes, were opened up to public use. At first, radio played only music. Then, gradually, stations began to broadcast dramas and comedies. It was not until the 1930s, however, that radio stations were running newscasts every day. Stations agreed to air the news for 5 min and tell stories that were no more than 12 hr old (Sweeting, 2015). During this period, radio access was highly uneven. In some American counties, virtually every household had access to a radio. In others, essentially nobody did. This affected politics: Counties with better access to radio were more likely to vote and received more generous relief funds during the New Deal (Strömberg, 2004).

The radio also influenced politics outside of the United States. During the 1920s, when Germany was democratic, the radio promoted a pro-democratic and anti-extremist narrative. Areas with better access to radio had lower levels of support for the Nazis. When Hitler came to power, this flipped. Messaging turned to pro-Nazi propaganda, and the effect reversed. Areas with radio access were more likely to support the Nazis (Adena et al., 2015). The role of radio in promoting conflict has been explored in a number of settings (DellaVigna et al., 2014; Gagliarducci et al., 2020; Straus, 2007; Wang, 2021; Yanagizawa-Drott, 2014).

Just as radio has been used to incite violence, it has also been widely used as a tool for social good. In many parts of the world, broadcast radio is the primary means of accessing news and information for millions of people. In Benin, for example, evidence from a natural experiment suggests that access to radio increased children’s literacy rates (Keefer & Khemani, 2014). In India, radio campaigns with stories discouraging people from supporting corrupt politicians led people to be less likely to vote for “vote buying” parties (Schechter & Vasudevan, 2023).

In Rwanda, psychologists and social scientists designed dramas to reduce prejudice and conflict (Paluck, 2009; Paluck & Green, 2009). The radio series portrayed a fictional story about two Rwandan communities that resembles the history and conflict between Tutsis and Hutus. In the drama, the community faces tensions about land-governance issues. As relations break down, the wealthier community is attacked. The violence creates victims, trauma, and refugees. However, some of the characters speak up against the warring leaders. The stories included educational messaging about prejudice, violence, trauma, and healing; they also promoted descriptive and prescriptive social norms in relation to intergroup behavior. The control group listened to an entertaining drama about reproductive health. Compared with listeners in the control group, treatment group listeners’ perceptions of social norms and their behaviors changed in a range of domains: intermarriage, open dissent, trust, empathy, cooperation, and trauma healing. Despite this, the treatment did not appear to change listeners’ personal beliefs with respect to intergroup violence.

In the United States, radio’s relative influence quickly faced pressure from television as people’s preferred source of media. Although people still listen to the radio in large numbers, television has become the primary mass medium by a considerable margin. People watch television for almost 3 hr per day on average—more than any other activity except sleeping and working (Bureau of Labor Statistics, 2022a). On a typical evening in the United States in 2021, roughly 35% of the population was watching television (Bureau of Labor Statistics, 2022b). The rise of television has had significant political impacts. For example, it is estimated that the growth of television caused somewhere between 25% and 50% of the total decline in voter turnout from the 1950s through the 1970s (Gentzkow, 2006). Moreover, the rise of Fox News is estimated to have led between 5% and 30% of non-Republican voters to switch their support to George W. Bush in the 2000 election (DellaVigna & Kaplan, 2007). Outside of the United States, exposure to West German television resulted in people in East Germany reducing their fertility rate, developing higher aspirations, and developing preferences for Western goods (Bönisch & Hyll, 2023; Bursztyn & Cantoni, 2016; Hyll & Schneider, 2013). Television also changed people’s core beliefs. People from areas more exposed to Western television tended to believe that effort, rather than luck, determined one’s success in life (Hennighausen, 2015).^11^

In the past decade, social media has become a major outlet via which people consume stories. Facebook, for example, has roughly 2.6 billion active monthly users. These sites differ fundamentally from previous technologies in that they enable bottom-up diffusion of stories. Observers first heralded this new technology as a major democratic innovation when social media was used by activists during the Arab Spring (Howard & Hussain, 2013). Since the 2016 U.S. election, however, social media has come to be seen as a source of divisive and polarizing content. Facebook’s algorithm at present appears to produce echo chambers in which people are less likely to see counterattitudinal content (Levy, 2021).

In summary, stories have played a central role in human culture throughout history and likely before. They have enabled societies to maintain cultural practices and traditions for thousands of years, as can be seen by the continued relevance of religious, dramatic, and philosophical texts written long ago. Stories are also intimately connected to technological developments. The explosion of literacy rates in Europe following the printing press was motivated by a drive to read the stories in the Bible. The impact of television and social media is mediated in large part through the stories that the technologies broadcast.

## Mechanisms of Narrative Impact

This article identifies three core characteristics of narrative. Stories are grounded in temporal events, contain goal-oriented agents, and entail causal sequences. Combined, these features make stories engaging and enable people to extract meaning from them. *Transportation* and *engagement* describe the audience being cognitively and emotionally immersed in the story world. *Identification* refers to the strength of the connection between the audience and story characters. *Meaning making* describes how people extrapolate from the causal models embedded in the story to their own decision problems. This section discusses each mechanism in turn.

### Transportation

Good stories transport people into the story world. Transportation refers to the state of being so immersed in a story that the audience can forget where they are (Green & Brock, 2000; Slater & Rouner, 2002). Stories that do this hold the audience’s attention, enabling them to filter out environmental stimuli so that they can allocate cognitive attention and emotional energy to the narrative. Transportation affects the audience in several ways (Appel et al., 2015). They lose track of time as they focus their attention on multiple possible narrative endings. They become mentally involved as they picture themselves in the scene of the events and construct vivid imagery regarding the narrative setting and characters. They are emotionally impacted as they connect with the plot and characters. In this section, we focus on several key mechanisms that elicit transportation: suspense, perceived realism, emotional flow, and enjoyment.^12^

#### Suspense

Narrative often deals with the vicissitudes of human life (for reviews, see Busselle & Greenberg, 2000; Potter, 1988). The suspense elicited by this captures the audience’s attention.^13^*Suspense* refers to the feeling of being excited or uncertain about what comes next, in anticipation of the outcome of the plot. There are several kinds of suspense: In one, the story outcome is unknown, and suspense is elicited by anticipation of *who*, *what*, or *how*; in another, the outcome is known because of preceding events, and suspense is elicited by the anticipation of *when* (Harmon, 2010). In other words, suspense can be invoked when the audience is deeply curious about what will happen next because they do not know the ending or when they know the ending but do not know how or when it will happen (Hoeken & van Vliet, 2000).

#### Perceived realism

Another mechanism for transportation, perceived realism, captures the audience’s judgment that the narrative world reflects the actual world; realism can directly impact positive evaluation of a story’s message by influencing whether the narrative seems reasonable (Cho et al., 2014). People are thought to be concerned with the perceived realism of a particular fictional context over and above the literal truth (Graesser et al., 2002). An audience may regard a story as unrealistic and confusing if the story world unnecessarily diverges from the actual world (e.g., humans have six arms without context) or the story seems incoherent (e.g., a character’s name changes without reason).

Cho et al. (2014) propose five characteristics of perceived realism. The first is *plausibility*, whether the story events portrayed could happen in the real world. The second is *typicality*, whether events are within the audience’s set of past experiences. *Factuality* refers to how much a narrative is perceived as portraying a specific individual or event in the real world. *Quality* refers to the degree to which the audio, visual, and other manufactured elements of a narrative evoke a convincing and compelling portrayal of reality, independent of whether the content of the narrative is related or relevant to the audience’s real-world experience. Finally, *perceived narrative consistency* denotes the degree to which story elements are judged to be congruent, coherent, and free from contradictions.

To illustrate, in one study, the protagonist in a story was described as lacking the ability to turn invisible but then disappeared (Walsh et al., 2018). Participants’ comprehension of the narrative was disrupted because the story’s fantasy context (i.e., that the character lacks the power of invisibility) and their general world knowledge (i.e., that people cannot turn invisible) were inconsistent with the target event (i.e., disappearing). Similarly, in a different study, anomalous passages (e.g., “Robert used a radio to play the horrible mouse”) tended to be more difficult to comprehend than implausible passages (e.g., “Robert used a hook to catch the horrible mouse”) and control passages (e.g., “Robert used a trap to catch the horrible mouse”; Joseph et al., 2008).

#### Emotional involvement, flow, and enjoyment

Emotional involvement is another key feature of transportation.^14^ Stories can act as a platform for people to suspend disbelief and vicariously pursue intense emotional journeys—they can make people burst into tears, cackle with laughter, or nervously slide back and forth in their seat, hoping for an alleviating turn of events (Nabi & Green, 2015). Emotions can immerse the audience in the plot to such an extent that they lose touch with their surroundings (Green et al., 2004). Evidence for the mediating role of emotion in narrative can be seen in a study by Morgan et al. (2009), which found that emotional involvement predicted beliefs about organ donation. Participants watched six episodes with organ donation storylines in four acclaimed U.S. television dramas (*CSI: NY*, *Numb3rs*, *House*, and *Grey’s Anatomy*). Greater emotional involvement was associated with stronger belief in the importance of organ donation, stronger perceived empowerment of other viewers to become donors, and participants’ beliefs that they had learned new facts about donation.

Emotions also help audiences comprehend story events. When consuming narratives, audiences regularly assume the perspective of the characters and mentally represent the characters’ emotional states as their own (Mar & Oatley, 2008). Such self-referent emotions are among the most direct means by which stories impact comprehension and motivation (Dunlop et al., 2008). Mentally representing the emotional states of characters requires that the audience can identify characters’ goals to guide interpretation of conflict and resolution in the plot (Oatley, 1999). In this vein, Levine and Pizarro (2004) suggest that emotions arise from event appraisal relative to the status of some goal. Positively valenced emotions (e.g., happiness) tend to be experienced when goals succeed and problem solving is no longer necessary. By contrast, negatively valenced emotions (e.g., sadness) emerge when goals have failed and there is a problem to solve.

Building on emotional involvement, emotional flow is another mechanism for transportation. *Emotional flow* refers to emotional shifts from positive to negative (e.g., happiness to sadness), from negative to positive (e.g., fear to relief), or from one state to another of the analogous valence (e.g., happiness to pride or fear to anger; Nabi & Green, 2015). The literature points to story structure as a key driver of emotional flow and transportation. Emotional flow is elicited by the dynamism of stories, produced by environmental and character changes, which take the audience on a journey through the ups and downs of the plot, including failures and successes. The plot defines the problem, establishing cause and effect between events that underly emotional shifts.

Narratives tend to converge on particular patterns of emotional flow. To illustrate, one study quantified the emotional peaks and valleys of more than 1,700 digitized novels and other texts (Reagan et al., 2016). Analysis revealed six essential emotional arcs that correspond to various plot archetypes: (a) rags to riches (rise); (b) tragedy, or riches to rags (fall); (c) man in a hole (fall-rise); (d) Icarus (rise-fall); (e) Cinderella (rise-fall-rise); and (f) Oedipus (fall-rise-fall). The tendency to construct plots that yield recognizable emotional arcs underscores the delicate balance between uncertainty and predictability. To this effect, age-old fairy tales, such as Cinderella and Little Red Riding Hood, have recognizable emotional arcs that have persisted despite variation in population histories and geographical distances across hundreds of years (Graça da Silva & Tehrani, 2016; Tehrani, 2013).

Another driver of emotional flow and heightened transportation is hedonic desire—people seek out messages that alter negative moods as well as maintain and prolong positive ones; this hedonic desire moves the audience to alternate between fear and hope as events progress in the story to make the protagonist’s goal more or less likely to succeed. This culminates in the cathartic experience of relief when the protagonist overcomes their obstacle. Hedonic desire generates postmessage transportation by driving people to seek more information, repeated exposure, recall, and social sharing. Negative information at an event boundary guides the audience to prioritize anticipation as they seek to shift their mood (Nabi & Green, 2015). Emotional flow can be so effective that even when a story outcome is unambiguously favorable, relief (negative to positive) mediates the effect of suspense on enjoyment (Madrigal et al., 2011). In one study, participants viewed film excerpts with multiple emotional shifts, wherein negative outcomes were emphasized (Bezdek & Gerrig, 2017). Attentional capture was measured by the participants’ reaction time to audio probes—lower reaction times conveyed greater transportation (i.e., failure to attend to external stimuli). Participants missed more probes and were slower to react during suspenseful scenes that signaled an upcoming emotional shift.

Thus far, we have considered how emotional involvement and flow work to enhance transportation. Yet another emotional driver of transportation is *enjoyment*, which refers to “a perception of great pleasure and happiness brought on by success in or simple satisfaction with an activity” (APA Dictionary of Psychology, 2023).^15^Nabi and Krcmar (2004) theorize that enjoyment comprises three dimensions that mutually reinforce one another to drive narrative consumption and transportation. The affective component relates to emotional flow, involving gratification-seeking and hedonistic desire as people ride the ups and downs of the narrative arc. The cognitive component involves judgments of characters’ actions, whether positive or negative, as well as judgments about the story content more broadly (e.g., perceived realism, story coherence, message quality) or personal evaluations (e.g., relevance, similarity). Finally, the behavioral component relates to selective exposure to the narrative based on the act of processing the narrative itself (e.g., reading vs. watching).

One way that enjoyment is relevant is through its ability to counteract the effects of fear (Moyer-Gusé, 2008). Often, communication that elicits high levels of fear discourages audiences from considering the message. This results in selective avoidance and story-inconsistent attitudes and behaviors (Moyer-Gusé, 2008). However, when a story is so enjoyable that it transports people into the story world, the audience often willingly experiences intense arousal, anxiety, and fear because the audience expects that the narrative will have an entertaining payoff (Zillmann, 1996). For example, people might enjoy the drama of thrillers or the visual effects of horror movies.

When people consume stories for enjoyment, they process information differently from when they consume information with the intention of learning. This can be seen in the results from a study that examined the effect of a biographical film on attitudes toward a political candidate (Weber & Wirth, 2014). Using voice-over narration, the study varied how a political candidate was portrayed (mildly positive vs. dramatically, exaggeratedly positive). The study also varied each participant’s motivation by giving them different instructions before the film began (to learn vs. to enjoy). Exaggerated portrayals yielded more favorable attitudes toward the candidate when the film was processed for enjoyment but not when processed for learning. The audience apparently tolerated story exaggerations less during didactic story comprehension because dramatic story content did not match their intention to learn.

### Identification

Good stories connect audiences to their characters (Cohen, 2001).^16^ This happens in several ways. First, people project the self onto the represented characters, a process termed *mentalizing* (Mar & Oatley, 2008). This enables people to take a character-oriented perspective, forming a bond between audience and characters (Murphy et al., 2011). The bond also guides the audience’s emotional response to events within the story: developing empathic feelings, understanding the character’s motives, adopting the character’s goals (Cohen et al., 2018), and unconsciously copying the behavior of the characters they observe (Lee & Shapiro, 2016). As characters push the plot forward, they increase the audience’s investment in narrative outcomes. Hence, well-fleshed-out narratives include intriguing characters with whom the audience can identify: Victims who suffer, villains who inflict harm, and heroes who vindicate the victims and avenge the villains. The best-documented ways to elicit identification appear to be based on characters’ perceived likeability (Robinson & Knobloch-Westerwick, 2017), similarity (Cohen et al., 2018; Hoeken et al., 2016), and point of view—that is, whose perspective guides the storytelling (de Graaf et al., 2012).

#### Likeability

Likeability is one known driver of identification (Robinson & Knobloch-Westerwick, 2017). *Liking* simply refers to positive evaluations of a character (Cohen, 2001). People seem to evaluate the likeability of media characters in much the same way they evaluate real people in their social networks (Mar & Oatley, 2008). That is, the audience assesses characters’ personality traits, developing impressions and expectations of characters’ behaviors. This increases the audience’s investment in the plot—people fear negative outcomes and hope for positive outcomes for liked characters and experience the converse for disliked characters (Zillmann & Vorderer, 2000).

One way to increase the likeability of characters is to provide recognizable features that cue schemas suggesting the characters’ morality (Krakowiak & Oliver, 2012; Tamborini et al., 2010). For instance, in one experiment (Grizzard et al., 2018), people interpreted visual cues about characters on the basis of schemas about heroes and villains (e.g., “ugly” villains dressed in dark clothes vs. “handsome” heroes dressed in pale clothes). This allowed participants to evoke character-consistent moral judgments even without reading about concrete behavior, characters behaving like a villain by doing harm, or characters behaving like a hero by helping. Moreover, the study found that character-schema activation was magnified by the presence of an opposing character (e.g., villain vs. hero), altering subsequent moral judgments of characters. The implication is that there is great power in suggestive cues to encourage the audience to imaginatively flesh out characters.

Nice characters are not always the most liked or most likely to yield identification; people are also attracted to negative characters (Hoffner & Buchanan, 2005). For example, moral, immoral, and morally ambiguous characters can influence audience responses in different ways. Krakowiak and Oliver (2012) found that good characters are well liked and thoroughly transporting. Morally ambiguous characters, in contrast, are liked less than good characters, but they are nevertheless equally as transporting, suspense inducing, cognitively engaging, and thereby enjoyable. The authors found that bad characters were liked the least but were equally as transporting, suspenseful, and thus cognitively engaging. Krakowiak and Oliver suggest that the audience may base their likeability of characters on the ratio of good to bad things that they do, particularly when lacking other information. In turn, this impacts identification.

#### Point of view

The narrative’s *point of view* refers to the perspective from which the story is told, whether first person, second person, or third person. Oatley (1999) suggests three reasons why point of view is crucial for identification. First, fiction involves mental simulation of other people’s minds, wherein coherence is determined by personal truths that come from a certain character’s perspective. As people simulate the experience of characters, the point of view provides scaffolding to connect to the character. Second, narratives have a constructive nature that does not always provide a faithful rendering of the events. It therefore matters for interpretation which ground truth is highlighted. Third, narratives enable people to conceive and understand goals, which necessarily relies on the point of view of the characters.

One of the main ways that point of view generates identification is to decrease the perceived cognitive distance between the audience and the character. Specifically, a first-person perspective helps the audience identify more strongly with the character’s experiences, aligning the audience’s feelings and attitudes with those of the narrator. Evidence for this comes from a series of experiments (de Graaf et al., 2012) that manipulated identification by varying story point of view. All participants read a narrative about a job interview for the position of web designer. One group read the version told from the applicant’s perspective. A second group read the version told from the perspective of the programmer who was hiring on behalf of an employer. Identification with the applicant mediated the effect of perspective on positive attitudes toward the employer. In a follow-up experiment, the narrative was about two sisters considering euthanasia for their mother, who had been in an irreversible coma for more than a month. Participants who read the story told from the perspective of the character against euthanasia identified more strongly with that character and held a less favorable posttest attitude toward considering euthanasia, compared with participants who read the story told from the perspective of the character who supported euthanasia.

#### Similarity

*Perceived similarity* refers to how much the audience perceives that they resemble a story character. Similarity can refer to physical attributes, demographic variables, beliefs, personality, or values (Cohen et al., 2018). A long-standing body of work posits that people are attracted to others who have similar identities and espouse similar attitudes (i.e., “birds of a feather flock together”; Byrne, 1971; Montoya et al., 2008). Other research supports this idea, finding that identification does correlate with self-reported perceived similarity (e.g., Hoffner & Buchanan, 2005). Similarity may also mediate romantic attraction to fictional characters, termed *parasocial attraction* (Andsager et al., 2006; Pinkleton et al., 2010). Although extensive research predicts that (demographic) similarity should predict identification (see Cohen et al., 2018, p. 508, for a review), more recent work has shown that basic demographic markers alone are insufficient to elicit identification (Cohen et al., 2018, Studies 1 and 2).

A well-documented way to elicit identification via similarity is to include self-referential details in stories; people preferentially identify with characters who appear not only similar but also relevant to themselves. For example, in one study (de Graaf, 2014), participants read a story in which the protagonist had either the same living arrangements as themselves or different arrangements (living with parents vs. in student housing). Participants with similar living arrangements displayed more story-consistent beliefs than participants with dissimilar arrangements. Yet this effect depended on whether readers related the story to themselves, not just identification with the protagonist. Equally, young participants who read a health testimonial identified more strongly with a young protagonist of the same gender than with an older protagonist of the opposite gender, but only when self-referencing mediated the effect (M. Chen et al., 2016).

### Meaning making

Meaning making describes how people extrapolate from the causal models embedded in the story to their own decision problems. Stories facilitate meaning making by supporting encoding of ideas and processing of important connections (i.e., causal junctures). Stories organize complex information into simplified causal structures. These are called *schemas* and *scripts* (W. F. Brewer & Lichtenstein, 1980). Schemas are general mental representations, depicting a concept’s parts and the relationship between the parts (Mandler & Johnson, 1977). Scripts are a related construct that convey temporal sequencing. They contain procedural knowledge about how events unfold: what happens and in what order (Schank & Abelson, 1977). Schemas and scripts are integral to narrative comprehension because people do not typically remember a narrative verbatim. Rather, they use schemas to retrieve the gist of the plot. One benefit of this is that people can flexibly recover information generalizing across other stories, subjects, and modalities (Baldassano et al., 2017).

For example, a children’s book may tell the story of a girl genius. After receiving admiration and attention from the adults in her life, she becomes hubristic and takes her friends for granted. Eventually, she realizes that her newfound self-confidence is in fact arrogance and has pushed her friends away. Her experience of loneliness forces her to see the error of her ways and she sincerely apologizes to her friends. Children reading this story can derive several sources of meaning. One is that hubris, though enticing, can isolate you from your friends. Another is that heartfelt apologies can be a path to redemption. These sequences of events are causally related, providing a practical schema that children can use to guide their social interactions. The schemas are not always obvious or explicit. Children are most effective at extracting these moral stories when prompted to explicitly explain the causal models embedded in the stories (Walker & Lombrozo, 2017).

#### Encoding

*Encoding* describes the conversion of information into representations that can be stored in the mind and recalled later from long-term memory (Goldstein, 2014). Schemas help people efficiently encode stories by providing preprogrammed structures in which novel information can be situated (Mandler & Johnson, 1977). This also facilitates retrieval (Black & Bern, 1981). Once a schema is cued, people regularly fill in the gaps with general knowledge or stereotypes without referencing the actual story (Schank & Abelson, 1977). For example, mentioning a restaurant automatically cues behavioral scripts related to dining, such as using cutlery and ordering from a menu, before the audience even encounters these concepts in the text. The importance of schemas for encoding is evident when stories violate expectations (Mandler & Johnson, 1977): Just the right amount of violation can heighten encoding because the audience tries to make sense of an unexpected event. By contrast, without any violation, the story is entirely predictable, not requiring encoding of diagnostic events for meaning making. Equally, too many violations can lead to confusion as the audience struggles to understand even basic story features (Barrett & Nyhof, 2001).

Another feature of stories that affects people’s capacity to encode is the cohesiveness of the narrative’s sequence. Well-organized events (e.g., beginning, middle, end) help the audience to understand how the story hangs together. The temporal connection between events helps people identify their underlying causal association: which events are connected or distinct, which are causal or peripheral (Kintsch, 1998). In turn, causally connected events have stronger associations at encoding (Black & Bern, 1981). To determine whether events are causally connected, the audience processes narrative text sentence by sentence, enabling them to observe which sentences refer to the same concepts and objects (i.e., establish referential relations). These relations signal associations between events, establish consistency, and facilitate detection of violations or anomalies (Joseph et al., 2008). Indeed, causal coherence is one reason why narratives are more readily recalled than expository information.

To illustrate, in one experiment, participants read narratives, then saw sentences from those narratives and tried to recall the sentences that came immediately after (Black & Bern, 1981). In a second experiment, participants engaged in free recall of the same narratives without cuing. In both experiments, recall was better when the two sentences were causally related. In free response, participants were more likely to recall two causally related sentences as one unit (as measured by conjunctions or summary statements). If one sentence was recalled, so was the other, and participants explicitly marked the connection between the sentences. These findings suggest that as the audience encodes one sentence, that sentence serves as a memory cue for encoding of the next one.

#### Causal junctures

Causal junctures aid meaning making because they leverage the causal logic of the story to convey which information is valuable and show possible ways to make sense of the story world (Dahlstrom, 2010). To investigate how the presentation of causal junctures affects the audience’s experience of stories, Knobloch et al. (2004) varied the attributes of stories that participants read, including the story’s causal chain (i.e., linear vs. reversed [out of order] vs. inverted [outcomes featured first]) and factuality (i.e., high [news reports] vs. low [novel excerpts]). The linear organization of events increased audience suspense while the reversed organization of events elicited more curiosity. Equally, the linear and reversed stories both produced greater reading enjoyment than the inverted story. These effects were independent of the factuality of media content, underscoring the value of meaningful connections between events. Indeed, the findings correspond with neural work showing that even the emotional experience of suspense depends on brain areas associated with predictive inference: Order helps people anticipate causal junctures at future event points (Lehne et al., 2015).

Another way that causal junctures drive meaning making is by indicating which information is relevant (Sloman, 2005). That is, causal junctures mark cause-and-effect relations between events, indicating which story elements are most likely to affect upcoming events. In one study (Dahlstrom, 2010), scientific assertions placed at causal locations of a narrative resulted in greater levels of acceptance of information than the same assertions placed at noncausal locations within the same narrative. Specifically, the information at causal locations was perceived as more truthful in the real world than the same information placed at noncausal locations. In a related finding, causally related events had greater impact when located at the beginning of the story, possibly because people dedicate more intense cognitive processing to anticipate the plot (Dahlstrom, 2012). Thus, it may be optimal to frame the sense of the story or convey more complex information at the beginning of the story, where story content receives most cognitive processing.

In summary, stories impact their audiences through three main mechanisms. First, stories are impactful when they transport people into the story world, capturing their attention and engaging them emotionally. Stories tend to transport people when they are suspenseful, when they are perceived to be realistic, and when they get the audience emotionally involved or interested. Second, stories are impactful when they lead people to identify with their characters. Three factors that matter for eliciting identification are likeability, the narrator’s point of view, and similarity. Third, stories have impact when their audiences are able to extract meaning from them. This means they can apply insights from the story to other contexts. People are best able to do this when the meaning, or schema, is easy to encode and when it is placed at causal junctures.

## The Functions of Stories

Stories have served a social function for thousands of years. Today, they aid a diverse array of goals—teaching children to read, persuading people to have safer sex, and inculcating national myths that bring polities together. This section brings these applications together under three headings: learning, persuasion, and collective action. *Learning* refers to how stories extend social learning and aid teaching. *Persuasion* describes how stories change people’s attitudes and beliefs by reducing reactance, conveying causal models, and facilitating vicarious engagement. And *collective action* relates to how stories address social dilemmas and coordination problems by establishing common knowledge, expectations, explanations, reputations, and shared identities.

### Learning

From early childhood, a central way people learn about the world is through story.^17^ People use stories to teach children how to read (Price & Kalil, 2019), as scaffolding to impart lessons on norms and morality (Baumeister et al., 2004; Walker & Lombrozo, 2017), and to explain how the natural world works (Dahlstrom, 2014). Stories are key to at least two information transmission processes: social learning and teaching. *Social learning* describes how people acquire knowledge through observation or interaction with other agents (Heyes, 2018). Stories extend social learning by enabling people to learn from others without directly observing the behavior (Baumeister et al., 2004). *Teaching* describes how a knowledgeable person intentionally facilitates the acquisition of information by a naive pupil (Galef & Whiten, 2017).^18^ Stories enable teaching by engaging their pupils and communicating causal models of the world.

#### Social learning

People learn to solve problems in two basic ways that are relevant here. One is trial-and-error learning. Imagine learning how to ride a bike. The other is social learning. People develop a vast array of their capabilities through the process of social learning (Henrich, 2017; Herrmann et al., 2007; Heyes, 2018; Tomasello, 1999). This is a key determinant of historical evolution and persistence (Henrich, 2020; Laland, 2018; Mesoudi, 2011; Nunn, 2020; Richerson & Boyd, 2005). Social learning was first theorized in detail by Albert Bandura in his work on aggression (Bandura, 1977). In Bandura’s original social learning paradigm, schoolchildren observed an adult model’s aggressive behavior toward a doll, a sequence that had certain storylike qualities. The children subsequently imitated the behavior of the adults. These experiments set the stage for understanding the much larger impact of social learning on behavior via goal pursuit, self-efficacy, and skill development.

One kind of social learning is *observational learning*. This describes an audience seeing others receive rewards and punishment for different actions and then flexibly shaping their own behavior on the basis of the observed strategies (Bandura, 1986).^19^ For example, one might see an older cousin take on a peculiar extracurricular activity and gain admittance to a high-quality university, then decide to take on that extracurricular activity oneself. Stories enable people to mentalize these experiences without ever having the actual social referents (Bandura, 2006; Baumeister et al., 2004). For example, one study tested whether watching the movie *Queen of Katwe* led Ugandan school children to perform better on their national exams (Riley, 2022). The movie depicts the struggle of a 10-year-old girl, Phiona, and her family, who live in poverty in the capital, Kampala. Her world is transformed when she meets a missionary who teaches her how to play chess. She soon discovers she is exceptionally talented, and her success in competitions enables her to escape poverty and buy a home for her family. Simply watching the movie improved both girls’ and boys’ performance in exams (compared with a placebo), but the effects were largest for girls. Girls were also more likely to continue their school after the exam; the movie entirely eliminated the gender gap in admittance to university.

The idea of stories as observational learning has motivated policy researchers to create narrative movies aimed at facilitating learning. In another study, a team of development economists traveled to rural parts of Ethiopia where people were living in poverty and had limited or no access to television. In randomly selected villages, they organized screenings of documentary-style stories depicting similar families getting ahead economically by working hard and making good financial decisions. The characters in the documentaries started businesses, diversified their income streams, and improved their farming practices. By setting goals and working toward achieving them, the protagonists improved their economic lot in life. The villagers who watched the documentaries were more likely to save money, use credit, enroll their children in school, and financially invest in their children’s education (Tanguy et al., 2014).

Observational learning is not mere imitation. The audience makes inferences about costs and benefits of actions on the basis of the model’s experience. Thus, stories may also lead people away from the behaviors they see modeled. An example of this is the impact of MTV’s television show *16 and Pregnant* on rates of teen childbearing (Kearney & Levine, 2015). In a particular region, an association was found between viewership of the show and changes in teen childbearing rates, suggesting that the show reduced teen births. To test whether the relationship was causal, the researchers employed an instrumental-variable strategy using local-area MTV ratings data to predict local *16 and Pregnant* ratings. The authors suggest that pregnancy rates may have fallen because of increased use of contraception and abortion, citing data from Google Trends and Twitter showing that the show increased interest in these search terms.

Stories are particularly helpful in learning how to navigate the social world (Dodell-Feder & Tamir, 2018; Mar, 2011; Mar & Oatley, 2008; Mar et al., 2006, 2009; Oatley, 1999; Tamir et al., 2015). Fictional stories simulate interactive experiences, activating parts of the brain used for social cognition (Tamir et al., 2015) and providing models of coordination (Mar & Oatley, 2008). They lay out the dynamics of human conflict. They describe the desires, frustrations, and obsessions of their protagonists. They portray acts of courage and betrayal. As people entertain fictional simulations again and again through the books they read and the shows they watch, people practice social interaction and develop more refined expectations for how social interactions play out (Oatley, 2016). One study (Mar et al., 2006) looked at whether different types of reading (i.e., fiction vs. nonfiction) predicted capabilities in social cognition. Reading more fiction predicted better social capabilities. In research on the short-term effects of reading fiction, participants were randomly assigned to a narrative condition, where they read stories, or to a control condition, where they read nonfiction or do nothing. The results were mixed. Some studies have found that reading fiction improves sociocognitive abilities (Kidd & Castano, 2013; Pino & Mazza, 2016). Other studies have found no effects (Panero et al., 2016; Samur et al., 2018). A meta-analysis of the relationship, examining evidence from 14 studies, found that these results are significant but small (Dodell-Feder & Tamir, 2018).

Stories may be effective social learning strategies for at least two reasons. The first is model availability (i.e., whom to observe). People are strategic social learners—they are highly selective in deciding whom to learn from (Hoppitt & Laland, 2013; Laland, 2004; Rendell et al., 2011). The advantage of stories is that they depict events that people rarely observe in ordinary life. For example, they might depict how a divorce plays out (an event that often happens privately) or how a person trains for a marathon (often alone, over time). The second is that stories are focal points for social coordination. Groups are capable of settling on a diverse set of social norms and moral lessons. The important consideration for group cohesion is not only the particular moral lessons learned but also the fact that everybody learns the same one. When people learn from the same stories, they converge on shared understandings.

#### Teaching

Stories are also important for teaching. A large literature shows that children whose parents read to them when they are babies and preschoolers are better able to read later (Bus et al., 1995). Historically, much of this research has been correlational. The large and widening class gaps in the time parents spend on developmental activities with their children (Altintas, 2016) create a risk that the association between reading to children and their cognitive development may be driven by other factors (such as financial resources). Some recent studies have been designed to address this. One study (Price & Kalil, 2019) undertook different methodological approaches to control for confounds. The study found that an increase in reading time (of 1 standard deviation) increased children’s reading achievement (by 0.8 standard deviations). One explanation why stories may be so key to learning how to read is that they are significantly easier to understand and remember than comparable forms of information. A recent meta-analysis by Mar and colleagues (2021), which examined 75 samples from more than 33,000 participants, found that people are significantly better at understanding and remembering stories than essays.

Stories are used to teach children other core skills, too. An example is the show *Sesame Street* (Kearney & Levine, 2019; Mares & Pan, 2013). *Sesame Street* focuses on teaching children how to be smarter, stronger, and kinder. The show began in the late 1960s with the goal of tackling educational inequality based on differences in access to quality preschool for disadvantaged children. It quickly became enormously popular. Scholars estimated that approximately a third of children in the United States between the ages of 2 and 5 watched the show in the early 1970s (about the same proportion of the U.S. population watches the Super Bowl today). Because of its reach, the show was radically more cost effective than other early childhood interventions. Early evidence from randomized trials revealed that the show had a significant and immediate impact on literacy and numeracy among children between 3 and 4 years old. The effects were comparable with those found in early Head Start evaluations (summarized by Kearney & Levine, 2019). *Sesame Street* has now been running for more than 50 years and is broadcast all around the world. A review of the impacts of the show in 15 countries, examining more than 10,000 children across 24 studies, found that the program had a significant positive effect on numeracy, literacy, health and safety knowledge, and social cognition (Mares & Pan, 2013). An analysis of the effects of broadcasting in the early 1970s (Kearney & Levine, 2019) examined variation in access to the show to estimate the effects, which were largest for children from disadvantaged neighborhoods as well as for boys and Black children. The show cost only $5 per child in 2019 dollars.

Stories play a role in teaching information to adults—this is sometimes called *entertainment education* (Singhal et al., 2003; Singhal & Rogers, 2012, 2002) or *infotainment*. One area of focus is financial literacy. In one study, researchers looked at the effect of embedding educational messages about debt management and gambling in a soap opera. The show featured a protagonist who borrows too much, gambles, and falls into a debt trap. Eventually, she seeks help to get out of her situation and manages debt responsibly. To test the effect of the show, the researchers (Berg & Zia, 2017) offered financial incentives as encouragement for two groups: One group watched *Scandal* (the show with the storyline about debt), and the other watched *Muvhango* (which screened at the same time). The overwhelming majority of the participants watched the shows they were assigned (< 12% of the control group watched *Scandal*). The researchers found that the show significantly increased financial knowledge, the use of borrowing through formal channels, and borrowing for productive purposes. The *Scandal* group were also significantly less likely to gamble. Focus groups indicated that the *Scandal* group emotionally connected with the protagonist and saw her make the kinds of decisions they might aspire to make.

### Persuasion

Narratives are also used to change attitudes, beliefs, and behaviors. Learning and persuasion differ in locus of control. Learning is about developing personal agency—the ability to “intentionally make things happen by one’s actions” (Bandura, 2001, p. 2). Greater agency means having more and better options to choose from or the ability to select between preferred choices at low cognitive cost. When people recover generalizable information from a story, through either observational learning or teaching, they enhance their capabilities and are better able to intentionally make things happen through their actions. Persuasion, on the other hand, is about influencing the beliefs, attitudes, and behaviors of other people. Persuasion may take a central route, where the target scrutinizes the merits of the information, or a peripheral route, where the target is influenced by superficial cues (Petty & Cacioppo, 1986), but the goal of persuasion is the same. When a story is used to persuade, the teller aims to affect the audience’s attitudes, beliefs, or courses of action. The locus of control lies with the persuader, not the audience.

Persuasion is widely used by policymakers around the world, though stories do not feature centrally in this work. In the field known as *nudging* (Thaler & Sunstein, 2008), governments create choice architecture that guides people to pay their taxes, encourages people to undertake healthy behaviors, and fosters more inclusive attitudes toward historically stigmatized groups. Many applications of persuasion have a paternalistic rationale (Thaler & Sunstein, 2003). For example, public health workers who want young adults to adopt safer sexual practices, such as using a condom, undertake this action because they believe that the targets of the policy will be better off as a result (Banerjee et al., 2019). Persuasion can also be a more cost-effective way to motivate action than legal punishment. For example, governments nudge people to pay their taxes as a compliment to traditional (more expensive) law-enforcement methods (Hallsworth et al., 2017). In these cases, the government would be acting in accordance with the law if it punished tax avoiders. Finally, the government may seek to instill civic virtues: attitudes, beliefs, and behaviors that are neither strictly in a person’s private material interest nor legally required. For example, government agencies may seek to discourage racist or sexist attitudes, or they may seek to encourage people to act prosocially within their community (Blair et al., 2019).^20^

There is a long-standing literature on narrative persuasion not just in social psychology but also in fields related to policy: communication (Braddock & Dillard, 2016; de Graaf et al., 2016; Moyer-Gusé, 2008; Moyer-Gusé & Nabi, 2010) and public health (Hinyard & Kreuter, 2007; Orozco-Olvera et al., 2019; Shen et al., 2015). Narratives have also been used to understand policy challenges such as intergroup conflict (Paluck, 2009; Tal-Or & Tsfati, 2016), outgroup prejudice (P. J. Johnson & Aboud, 2017; D. R. Johnson et al., 2013; Martinez et al., 2021; Moyer-Gusé et al., 2019), climate action (Morris et al., 2019), and trust in government (Trujillo & Paluck, 2012).

#### Attitudes and beliefs

Persuasion is first and foremost about changing people’s attitudes (Crano & Prislin, 2006) and beliefs (Kamenica, 2019). *Attitudes* describe how people evaluate targets with favor or disfavor (Eagly & Chaiken, 1993, p. 1). The target that people form attitudes about could be anything—actions, a group of people, or even the self. Beliefs, on the other hand, are expectations about the likelihood of different states of the world (Fishbein & Ajzen, 1975; Manski, 2004; Ramsey, 1931/2016). Attitudes and beliefs matter for policy for several reasons, the main one being that, under certain circumstances, they predict behavior. In their work on behavioral change, Fishbein and Ajzen (2010) model attitudes (alongside the person’s perception of social norms and behavioral control) as one of three psychological variables that determine behavioral intentions, the primary antecedent of behavior. They propose that beliefs, in turn, determine each of these variables. Policymakers may also be concerned with attitudes and beliefs for their own sake. For example, they may be concerned about the spread of fake news, the prevalence of prejudice or hate, general levels of distrust, or people’s mental health, viewed as their attitude toward themselves and their lives.

A powerful example of the capacity for stories to navigate sensitive and complex social attitudes comes from a remarkable study on female genital cutting. In Sudan, female genital cutting is prevalent, but social attitudes vary within communities (Efferson et al., 2015). In one study (Vogt et al., 2016), a series of movies portrayed the local variation in views on cutting. The movies depicted an extended family in a rural part of Sudan—parents, grandparents, children, and other relatives—and contained intrigue, deception, love, and forgiveness. The treatment conditions were embedded in a subplot in which characters have a disagreement in relation to cutting. One subplot focused on arguments about cutting, purity, health, and religious values. Another subplot focused on the effect of cutting on young women’s marital prospects. The movies significantly improved viewers’ implicit attitudes toward uncut girls compared with a control movie (which had no discussion of cutting). And the movie that combined both subplots had relatively persistent effects.

The best holistic assessment of the effect of narrative persuasion comes from a meta-analysis of 76 studies conducted between 1983 and 2013, which found that narrative interventions have a significant effect on attitudes and beliefs (Braddock & Dillard, 2016). There are several explanations for the persuasive effects of narrative: They reduce reactance, they supply causal information, and they expose their audiences to vicarious experiences.

#### How stories persuade

The first way in which stories persuade is that they reduce reactance (Moyer-Gusé, 2008; Slater & Rouner, 2002). *Reactance* describes audiences feeling that a message threatens their freedom or pressures them to change. This experience may lead people to be more likely to counterargue (Brehm & Brehm, 2013). Stories can be designed to reduce counterarguing by embedding persuasive messaging in an engaging plot without making the audience feel that they are the target of the message. For example, a story may include a plot in which one character is about to make a bad health choice and another pleads with them to consider the consequences. In doing so, the story exposes the audience to the argument without ever making them feel that the story is explicitly seeking to persuade them. In turn, the audience may also focus on how the recipient of the message responds within the story. A recent meta-analysis found that narratives were more effective than nonnarrative persuasion at reducing counterarguing and that story engagement (discussed in an earlier section) predicted the degree of counterarguing (Ratcliff & Sun, 2020).

Second, stories represent *causal relations* that people then use to make sense of the world (Dahlstrom, 2010; Eliaz & Spiegler, 2020; Kendall & Charles, 2022). For example, a fictional story about corporate lawyers may describe how the characters, employed at top law firms, got their jobs through connections rather than grades. The audience, aware that the story is fictional, knows that this information does not pertain to actual events that happened. Nevertheless, the causal model embedded in the narrative (connections lead to job offers) may lead the audience (e.g., prospective law students) to update their beliefs about how they should spend their time at law school. The persuasive feature of the story in this pathway is not the information contained within the story but the mental model that offers a new way to organize existing information (Schwartzstein & Sunderam, 2021; Trabasso & van den Broek, 1985).

Third, stories facilitate vicarious experiences—for example, the experience of engaging with members of outgroups. One study (Moyer-Gusé et al., 2019) exposed mostly White and Asian samples of American participants from a midwestern university to a television show about a Christian man who lives with a Muslim family for 30 days and follows their customs. In the show, the man is apprehensive about living with the family and expresses concern about their views. Over the course of the 30 days, he comes to learn more about their community and benefits from interacting with them. The researchers compared this condition with a control condition that involved a show from the same series about a wealthy family who tried to live on minimum wage for 30 days. The treatment led participants to hold more favorable attitudes toward Muslims both immediately after the intervention and a week later. Mediation analysis indicated that participants who identified with the Christian character were more likely to feel capable of having conversations with Muslim Americans. This, in turn, predicted lower rates of social anticipatory anxiety and prejudice. An alternative route is to use narratives to target the prototypes that people hold of outgroups. A similar study exposed participants to counter-stereotypical Muslim exemplars. The study reduced intergroup anxiety as well as explicit and implicit prejudice (D. R. Johnson et al., 2013).

#### Persistence and rigidity

One question is whether the persuasive effects of stories quickly fade out. Evidence suggests that, at least in the short to medium term, the opposite happens: Effects may increase over a week or two. In one study, participants were randomly assigned to read a fictional narrative excerpt about a kidnapping or a nonfiction control story (Appel & Richter, 2007). Participants were asked to rate both the extent and the certainty of their agreement or disagreement with fact-related assertions they had encountered in the narrative. Half of the participants completed their responses immediately after reading the narrative, whereas the other half answered these questionnaires 2 weeks later. For the experimental group, encountering false assertions lowered the endorsement of previously held (true) beliefs, whereas encountering true assertions neither raised nor lowered belief endorsement. Changed beliefs were held with a higher certainty after a 2-week period.

Relatedly, once people have been exposed to stories about a group, the beliefs and attitudes shaped by those stories can be difficult to change. Portrayals become sticky. People pay more attention to and later remember stereotypical information about real or artificially created groups (Bratanova & Kashima, 2014; Judd et al., 2005). This bias for stereotypical information affects the transmission of stereotypical traits in chains of connected participants exposed to stories. For example, even in contexts in which participants remember stereotype-inconsistent information better than stereotype-consistent information in individual recall tasks, chains of connected participants recalling stories produce a reliable bias for stereotype-consistent information (Kashima, 2000). This bias could be due to people’s preference to discuss shared information, relative to unshared information (Stasser & Titus, 1985). And because stereotypical information could be assumed to be shared, this creates the circumstances for stereotypes to take hold following the dissemination of stories through social networks.

#### Behaviors

There are two ways in which stories are thought to influence behaviors. The first pathway is through changes to beliefs and attitudes, just discussed. People may formulate intentions to undertake a behavior in response to updating their beliefs about a target behavior, their perceptions about the social norms surrounding the activity, or their confidence in their ability to competently complete an action (Fishbein & Ajzen, 2010). An alternative route is automatic. People may mimic behaviors without ever consciously realizing that they do so (Zhou et al., 2017).

Experimental evidence of the effect of stories in real-world contexts has begun to emerge. In a randomized controlled trial conducted in Nigeria (Banerjee et al., 2019), roughly 5,000 young people were invited to watch soap operas, and the researchers examined their effect on sexual health behavior. In one condition, participants watched the television show *Shuga.* The show depicted young Africans from different social classes balancing their bright aspirations with the harmful consequences of high-risk behavior. In another condition, participants watched a show called *Gidi Up*. The show had a similar setting but no content on health. The researchers presented the movies in 80 sites across southwest Nigeria. Eight months later, participants in the treatment group were twice as likely to get tested for HIV than the control group. Participants were also more knowledgeable about HIV: They were more likely to know about its transmission and about antiretroviral drugs. The show did not increase self-reported condom use. However, the likelihood of testing positive for chlamydia fell by 55% among women in the sample. This may be because people decided to have fewer partners.

One consideration with studies such as this is that the stories may simply be efficient conduits for information. Could the outcomes of the soap opera have been achieved with a simple public service announcement? Research is still in the early stages. One study measured behavioral outcomes and compared the effect of narrative and informational videos on low-income African American women’s use of mammography, as well as their cancer-related beliefs, recall of core content, and range of reactions to the videos (Kreuter et al., 2010). Women from St. Louis, Missouri (aged 40 and older), were randomly assigned either to watch a narrative video containing stories from African American breast cancer survivors or to listen to equivalent informational content delivered in a lecture format. The researchers tested effects immediately after the video and also after 3 and 6 months. The narrative video raised women’s perception of the importance of cancer screening and led them to see mammography as a more effective way of protecting against the disease. The narrative video was most impactful for women with less than a high school education: 6 months later, this group was twice as likely to have gotten a mammography exam.

### Collective action

Finally, in addition to their uses for learning and persuasion, stories are key to managing collective action problems—namely, challenges characterized by interdependence. Political theorists emphasize that collective action is hard because individual decision-makers must make group-level choices about public matters (R. Hardin, 1982), in which people often have competing interests and often do not know what others believe or want. Social scientists and psychologists have long emphasized the role that stories play in collective action—driving the formation and dynamics of nations (Tilly, 2002), religions (Dunbar, 2022), hunter-gatherer communities (D. Smith et al., 2017; Sugiyama, 2001), organizations (Boje, 2008), cultural groups (Michalopoulos & Xue, 2021), and even financial markets (Shiller, 2017).

**Fig. 6.**
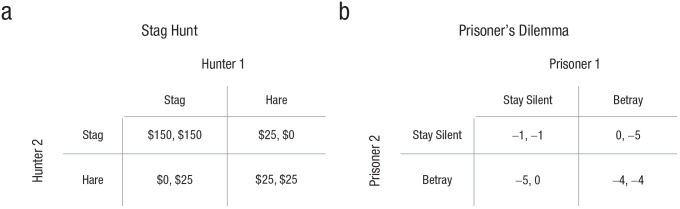
Coordination challenges and social dilemmas. The stag hunt and the prisoners’ dilemma are classic economic games. It is noteworthy that they are presented as vignettes or stories. The payoff structure for each hunter in the stag hunt is shown in (a), where the values in each cell correspond to Hunter 1’s payoff followed by Hunter 2’s payoff. The optimal strategy for each player depends on the actions of the other player. If Hunter 1 hunts stag, Hunter 2 will maximize their payoffs by also hunting stag. And if Hunter 1 hunts hare, Hunter 2 will maximize their payoffs by also hunting hare. Because the payoff for hunting hare is unconditional (i.e., it is not based on the decision of the other hunter), this is the risk-dominant strategy. Because the payoff for hunting stag is greater (i.e., it is larger than for hunting hare), this is the payoff-dominant strategy. The payoff structure for each prisoner in the prisoner’s dilemma is shown in (b), where the values in each cell correspond to Prisoner 1’s payoff followed by Prisoner 2’s payoff. As a group, the best course for the prisoners is staying silent, which will result in each getting only 1 year in confinement. But as individuals, both prisoners are better off betraying the other no matter what the other prisoner does. If Prisoner 1 stays silent, Prisoner 2 can get off entirely by betraying them. If Prisoner 1 betrays Prisoner 2, Prisoner 2 will still reduce their own sentence by reciprocating the betrayal. When the prisoners act in their private interest, they end up with the largest combined jail sentence.

To show how stories are used in collective action, it is necessary to describe the mechanics of two important collective action problems: coordination challenges and social dilemmas. *Coordination challenges* are situations in which the relative payoffs from one person’s actions are affected by others’ actions. Some kinds of coordination, such as deciding which side of the road to drive on, are simple. It matters little whether you drive on the right or the left side of the road as long as everybody else obeys the same rules. But many coordination challenges are more complicated. For example, collaborating may come with large payoffs, but only if everybody chips in. This scenario is described by the stag hunt (Lewis, 1969; Skyrms, 2001, 2004), a classic economic game in which it is in people’s interest to collaborate, but only if everybody else does so, too (see Fig. 6a).

*Social dilemmas* are situations in which it is in people’s shared interest to cooperate but in individuals’ private interest to “defect” (Dawes, 1980). This describes many of the world’s most urgent social problems—climate change, taxation, waste management, and public-resource use (Ostrom, 1990). Because theory predicts that these scenarios lead to collective failure, they are described as the “tragedy of the commons” (G. Hardin, 1968). The simplest case is captured in the two-person prisoners’ dilemma—the story of two prisoners who have been placed in separate interrogation rooms on suspicion of armed robbery. The police lack evidence to convict them of the armed robbery but found them in possession of illegal firearms, for which they can each get a 1-year sentence. The detectives separately offer each prisoner a deal:

We have you on illegal possession of firearms—that’s a 1-year sentence. We can do you a deal, though, if you testify against your coconspirator. They will get 5 years, but we’ll let you off. Now, that assumes they don’t testify against you! If they give evidence against you and you give us nothing, you’ll get 5 years. Either way, you’re better off testifying. If they testify against you, your testimony can still reduce your sentence. You’ll each get 4 years.

**Fig. 7.**
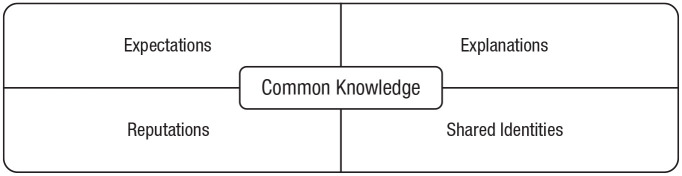
How stories affect collective action. *Expectations* describe the beliefs that people hold about the kind of interaction they are having, as well as their role and others’ roles within that interaction. *Explanations* describe peoples’ understandings of the systems they engage with (e.g., the economy, society, and the physical world). *Reputations* describe how people use others’ track record of behavior to decide how to interact with one another. *Shared identities* describe how people see themselves as members of groups—which, in turn, determines their social preferences. *Common knowledge*—which essentially describes when everybody knows they are all on the same page—is at the heart of all of these drivers of collective action challenges.

Collective action problems have well-understood solutions. The government can mandate cooperative behavior through threat of violence (Fukuyama, 2011), a foundational argument made by Thomas Hobbes (1651/1991). Alternatively, communities can leverage repeated interaction. When people know that they are going to be dealing with others again and again, they generally forecast that it is in their best long-term interest to cooperate (Axelrod & Hamilton, 1981). Communities can also engage in voluntary punishment. When groups can impose costs on defectors, they are able to sustain higher levels of cooperation (Fehr & Gächter, 2002). We add stories to this list. Stories affect collective action by establishing common knowledge, expectations, explanations, reputations, and shared identities (see Fig. 7).

#### Common knowledge

Groups generally require common knowledge to solve collective action problems (Lewis, 1969), and stories are an effective way of establishing it. *Common knowledge* means that members of a group all hold a particular set of beliefs and also know that the other members also hold those beliefs. It can be contrasted with mere mutual knowledge, where each person holds the knowledge, but no one is aware that others also hold it. To illustrate why common knowledge is important, imagine you are driving in rural Thailand, where people drive on the left-hand side of the road. You are close to the border with Cambodia, where people drive on the right-hand side of the road. The road you are driving on is barely wide enough for two cars, and the marking down the middle has faded. As you navigate the winding road, an oncoming car (the first you have seen in this borderland region) comes speeding toward you. You are certain that you are supposed to drive on the left-hand side of the road. The driver coming toward you also knows this, but you do not know that they know. They could be a tourist or a local who follows a different custom. After all, they are coming from a region that drives on the right. The road is narrow, and each car will need to shift left or right to get by. What should you do? Although both you and the other driver hold the correct knowledge, it is not enough. Your knowledge, though correct, is siloed. To avoid a crash, you will both need to signal your intention to each other to drive on the left (or right—again it does not matter as long as you make the same choice!).

Stories establish common knowledge in two ways. First, they spread information virally through networks—for example, by word of mouth, text, or social media. The following experiment offers a nice demonstration (Mesoudi et al., 2006): People shared information in four-person chains, a process like the game of telephone. Participants in the first round read information and passed it on to a second person. That second person passed it on to a third person, and the third person passed it on to a fourth. The research team afterward recorded the amount of information each person recalled and whether the recollection was accurate. To test what kind of information spread with the best strength and fidelity, they randomly varied the information they gave participants in the first round. One group received factual nonnarrative information about the city of Denver, Colorado. Another group received basic narrative structure but nothing remarkable—simply a description of ordinary events in a woman’s life. A third group received a prototypical story—gossip about a woman who had a sexual relationship with a married professor and became pregnant. Each paragraph contained the same number of propositions (defined as “a predicate plus a series of ordered arguments”; Mesoudi et al., 2006, p. 411) and was roughly the same length—thus, the informational structure was largely equivalent. But as the information ran through the chain, people recalled more propositions and recalled them more accurately in the gossip condition than in the other conditions. The prototypical story lived longer.

The first-order implication of stories going viral is that more people are likely to be exposed to information. But the second-order implication is more interesting. Virality also signals to the audience that other people have been exposed to the information. People want to know what others know. They are sensitive to being left out of the loop (Jones et al., 2009). When people know that others have seen and approved of particular viewpoints, they are more likely to adopt those viewpoints themselves (Vlasceanu & Coman, 2022). One reason for this is that when stories propagate extensively among individuals, for example, through conversations, the communities converge on the conveyed beliefs and intentions (Vlasceanu et al., 2018). In studies on this process, participants read stories and then individually recalled them, after which they engaged in several rounds of joint recollections as part of conversational social networks. Finally, participants once again recalled the initially studied stories. A burgeoning literature shows that communities composed of more interconnected subgroups converge faster on the same information if they interact soon after exposure to a public event, compared with communities of less interconnected subgroups (Momennejad et al., 2019). Furthermore, increasing people’s motivation to relate to one another during conversational interactions further accelerates convergence processes, as do people’s perceived similarity with one another (Coman & Hirst, 2015).

Why does this happen? First, people’s memories and beliefs are highly malleable (Chater, 2018; Schacter, 1999). This is what allows, under certain circumstances, alignment to occur following social interactions (Coman et al., 2009). The fact that previously encoded memories get strengthened on retrieval, for instance, indicates that the cognitive system maintains fluid mental representations that are likely to change over time, depending on circumstances. Second, social-influence processes manifested in social interactions impact the degree to which people’s cognitive representations become aligned (Coman & Hirst, 2015). As an example, the motivation to relate to one another in social interactions meaningfully influences how much people alter their memories and beliefs (Echterhoff et al., 2009). And third, synchronization among individuals at a local level leads to the emergence of collective memories and beliefs at a community level (Coman et al., 2016).

But stories may propagate in unexpected ways. Because stories are culturally dependent, their propagation relies on the ability of communities to synchronize. Stories both reflect and generate culture. That is, cultural dynamics circumscribe what people attend to, remember, and are willing to communicate to one another. These differences are showcased by a recent investigation into the generation of narratives in response to listening to instrumental music (Margulis et al., 2022). Participants in three different geographical locations (Arkansas; Michigan; and Dimen, China) listened to instrumental music and generated narratives to represent several musical excerpts. Natural-language-processing techniques assessed the similarity of these narratives within and across cultures. A clear pattern emerged: Participants from the same culture (Arkansas and Michigan) produced more similar narratives than participants from different cultures (Arkansas and Dimen; Michigan and Dimen).

What are the mechanisms for such a pattern? Psychological research points to two interrelated explanations: initial perception and subsequent filtering through cultural schemas. First, culture impacts the way information is initially processed. For instance, exposure to the same visual stimuli resulted in differences in information processing across different cultures. Japanese participants processed visual scenes more holistically, focusing on the relations among the different elements, compared with American participants, who employed item-specific processing (Masuda & Nisbett, 2001). Closer to a narrative instantiation of these differences, American participants segmented visual scenes of routine activities in more fine-grained ways, compared with Indian counterparts, providing evidence of Americans’ preference for analytic processing (Swallow & Wang, 2020). The source of these cultural differences so early on during the information-processing chain is speculative at best. One proposal is that they emerge because of cultural heterogeneity in early socialization practices and exposure to different environmental conditions that involve routine engagement in tasks that strengthen these preferences (Gelfand et al., 2011). Second, cultural schemas impact the way stories are processed and told (Karsdorp & Fonteyn, 2019). These schemas are defined as widely shared knowledge structures that provide default assumptions about an event’s characteristics and relations to other events (DiMaggio, 1997; Fiske & Linville, 1980). Through these culturally grounded cognitive schemas, a person can impose meaning on ambiguous information (Bartlett, 1932).

Stories also establish common knowledge from the top down, through mass media. Super Bowl ads are an illustrative example (Chwe, 2013). These ads often sell prestige goods such as cars and technology products—things that are valuable in part because they signal social status to others (Veblen, 1899). The point of these ads is not simply to reach a very large audience but also to signal to audiences that others are watching. The common knowledge produced by these ads can increase the status of these goods, establishing immediately that everybody knows about them and has seen them in a particular attractive light. Although to the best of our knowledge, nobody has systematically categorized the share of media content that is narrative and nonnarrative, stories make up a significant share of media content—for example, television series, movies, the news, reality television shows, and documentaries.^21^

One explanation for the effects of soap operas on fertility in Brazil, discussed earlier, is that the shows’ popularity established common knowledge about possible alternatives for families. An extensive literature in social science describes how individuals and families navigate complex social-expectations-related gender roles and family structures (Andrew & Adams-Prassl, 2023; Carvalho, 2013). Social practices are not simply an agglomeration of private interests. Rather, people act in anticipation of what others will think (Bicchieri, 2005; Bursztyn et al., 2020).

#### Expectations

Expectations are key to collective action because outcomes are jointly determined. Social interactions are generally complex, requiring that people, in real time, anticipate how others will act and how their counterparts will interpret and respond to their own actions. The literatures in psychology and social science emphasize the narrative quality of human action (Ostrom et al., 2002; Sarbin, 1990; Schank & Abelson, 1977). People learn how events play out through direct observation of other people (Bandura, 1977), but these learnings are incomplete. People also learn how to interact through the stories they hear as children from their parents, through the gossip heard about how others behave, and through the stories in mass media (Baumeister et al., 2004; Swidler, 1986; Zerubavel, 2009). Stories establish expectations about two important characteristics required for interaction. First, they define the interactive context (e.g., the characters are at a restaurant), which enables people to simulate how events will unfold (S. G. B. Johnson et al., 2022). Second, they signal to people their role in that context (e.g., the characters are dining), helping them identify which script or performance to act out (Bicchieri, 2005; Geertz, 1973).

Consider first the role of stories in establishing context. In an illustrative study, psychologists invited participants to play the prisoner’s dilemma (see Fig. 6b). One group of participants were told they were playing “the Wall Street game.” The other group were told they were playing “the community game.” Although all participants were presented with the same incentive structure, the labels evoked competing stories. Wall Street evokes narratives of greed, where the actors are self-interested. Community evokes narratives of cooperation, where the actors help each other out. If such stories were mere pageantry, participants would behave in roughly the same way regardless of the label the researchers put on the game. Yet roughly 70% of participants who were told that they were playing the Wall Street game chose to betray the other prisoner, whereas the proportion was the inverse in the community game. Among this group, roughly 70% of participants chose to cooperate (Liberman et al., 2004).

Next, consider how stories signal people’s roles to them. It is through the stories they watch and hear growing up that people learn what it means to be a good parent, worker, or citizen. The roles then affect decisions. One indicator of this is that when prisoners and finance professionals are primed with their identity, they behave more dishonestly (Cohn et al., 2010, 2014). Roles affect groups’ capacity for coordination by signaling who will undertake what action (de Kwaadsteniet & van Dijk, 2010). People know what is expected of a father, a lawyer, and a waiter (Biddle, 2013) and often accept the status and privileges associated with others’ role allocations because of their function in solving coordination challenges for the group (Clark et al., 2006; Keltner et al., 2008).

Roles exert a powerful influence on people’s capabilities and resources (Keltner et al., 2008). Moreover, because social roles are interdependently determined, they are difficult or impossible for individuals to change independently. It may be necessary for change to occur at the group level. Stories can be a powerful way for groups to negotiate new social roles. A study from India is instructive. Hoff et al. (2020) examined the effect of participatory theater on gender dynamics within the household. In the performances, the theater group enacted oppressive relations, then repeated the performance to enable members of the audience to take the role of protagonists and victims to address the oppression. This enabled groups to analyze the oppression and to explore ways to resist and change roles. Across 3,000 households in 87 villages, compared with villages that have never been exposed to the theater, women were significantly more likely to participate in decision-making roles and less likely to be part of abusive marriages. Studies have found similar results from increased exposure to cable television in rural India (Jensen & Oster, 2009).

#### Explanations

Collective action often requires that people converge on consistent explanations—accounts of why things happened the way they did. To enact laws to protect against the risk of another financial crisis, legislators must share at least a coarse explanation for why the financial crisis happened. To assess whether a defendant is guilty of murder, a jury must often agree why the defendant was behaving the way they were. For this reason, the policymaking process and the jury-based law system are often characterized not only by debates over facts but also by the narrative interpretations of those facts (Mukand & Rodrik, 2018; Pennington & Hastie, 1992).

Stories are central to the explanations humans formulate of social behavior (Bruner, 1991; Dennett, 1987, 1988; Sarbin, 1986, 1990). In a recent study on the nature and origins of people’s narratives about the macroeconomy (Andre et al., 2022), a series of broadly representative surveys of 8,000 Americans and 100 experts were created to investigate how people make sense of the genesis of inflation. Policymakers express more complex and abstract narratives such as loose monetary policy, whereas households are more likely to invoke politicized narratives about incompetent policymakers and greedy corporations. People’s explanations affect their expectations: Those who attribute inflation to the energy crisis or government mismanagement think inflation will last longer than those who attribute it to the opening of the economy after the COVID-19 lockdowns.

Narratives are often deployed in competition with one another to shape our interpretations of events. For instance, in the jury-based law system, trials typically entail debates over narrative interpretations of events. In essence, defense attorneys and prosecutors attempt to impose their story onto jurors. As an example, consider the case of Trayvon Martin, a 17-year-old African American high school student murdered by George Zimmerman, a neighborhood watch coordinator in a gated community in Florida. During the murder trial, Trayvon was described as either an innocent adolescent or a dangerous young adult. Elements supporting these narratives were carefully presented by the prosecutors (e.g., Skittles found in Trayvon’s pocket) and defense attorneys (e.g., prior high school suspension for possible marijuana possession), respectively. Pennington and Hastie (1992) proposed the *story model* of judicial decision-making to describe how this process of narrative construction and adoption could impact people’s decisions. According to this model, the jurors play an active role in the story-generation process as they reach a verdict by connecting different pieces of evidence and creating a causal structure for these events. They do so, this work indicates, by relying on three sources of knowledge: the evidence presented during the trial, their idiosyncratic knowledge of similar events, and their expectations about what makes a story complete. For judicial systems that involve juries, the processes that involve narrative construction are social. That is, the narrative-generation process, the connections among pieces of evidence, and the knowledge that the jurors bring to bear is constantly negotiated in social interactions among the decision-makers.

#### Reputations

Another common strategy for managing social dilemmas is to cooperate with others as long as they cooperate with you (Fehr et al., 2002; Fischbacher et al., 2001; Trivers, 1971). A classic example is the tit-for-tat strategy. This happens when actors repeat each other’s actions. For example, in an economic game, if Player 1 acts prosocially toward Player 2, Player 2 will then behave prosocially to Player 1. If Player 1 acts antisocially to Player 2, Player 2 would then reciprocate with an antisocial response to Player 1. Tit-for-tat strategies enable groups to converge on cooperative equilibria (Axelrod, 1984). Groups that expend more effort and cost to monitor and cooperate conditionally more effectively manage their common pool of resources (Rustagi et al., 2010).

A limitation of this approach is that people often lack firsthand knowledge of others’ prior behavior. One of the main ways that groups hold people accountable for their track record is by disseminating reputational information about them (Greif, 1989, 1993). People, as it turns out, are highly sensitive to the reputational consequences of their actions (Barclay, 2010; Haley & Fessler, 2005). Reputations mean that people’s track record can be used even when their prior behavior is not directly observable to their counterparts (Dunbar, 2004). Reputational information is most commonly spread through gossip, namely positive or negative evaluations of other people not present (Foster, 2004; Haviland, 1977). Gossip’s defining feature is its evaluative function; it does not exclusively take narrative form. However, stories are an important means through which people evaluate people’s characters, and people take care to craft narratives to shape impressions (Kim & Crockett, 2022).

Gossip may be even more effective than punishment at promoting cooperation. A multiround public-goods game gave people the option either to gossip about their partners (the ability to send notes to future counterparts’ future partners) or to punish them (take away resources from them with a fine-to-fee ratio of 3:1). Contexts in which people were able to gossip had more robust effects on cooperation than contexts in which people were able to punish. These effects persisted beyond the game. The research team then asked participants to play trust games after the public-goods games, and they found that participants in the gossip condition were more trusting and trustworthy. In line with this, psychologists and economists have examined the effect of reputation on collective action challenges (Milinski, 2019). They find the mere possibility that others may gossip and spoil their reputations leads people to behave more generously in dictator games and more prosocially in one-shot public-goods games (Beersma & Van Kleef, 2011; Piazza & Bering, 2008). People often rely on gossip even when direct observations of prior behavior is available (Sommerfeld et al., 2007).

#### Shared identities

Identity also affects groups’ capacity for collective action (Akerlof & Kranton, 2000, 2005). Social identity determines the boundaries of community membership (M. B. Brewer, 1991) and establishes people’s role within their group (Biddle, 2013). Group membership makes collective action easier because shared identity fosters prosociality and trust (M. B. Brewer, 1999; Y. Chen & Li, 2009; Wit & Wilke, 1992).

Stories play a key role in personal identity formation (McAdams & McLean, 2013). But stories are also important for establishing shared identities (A. D. Brown, 2006; R. M. Smith, 2003). Consider the nation as an example. Nations are perhaps the most important modern political unit. By providing the sociocultural underpinnings of the state, they determine where people can travel and work, as well as what other economic benefits they are entitled to enjoy (Fukuyama, 2014). Social scientists have converged on the view that nations are social constructions: “imagined communities” brought together by myths of commonality (B. Anderson, 2006). Psychologists, political scientists, and historians have addressed the role that origin stories play in the formation of national identities (Roediger, 2021; R. M. Smith, 2003; Tilly, 2002; Wertsch, 2021). According to R. M. Smith (2003), narratives of peoplehood

work essentially as persuasive historical stories that prompt people to embrace the valorized identities, play stirring roles, and have the fulfilling experiences that political leaders strive to evoke for them, whether through arguments, rhetoric, symbols, or “stories” of a more obvious and familiar sort. (p. 45)

The power of narratives is reflected in their centrality in politics. Origin stories often define the nature of the nation—its aspirations, values, commitments, and ultimately its integrity. Arguably, one of the central American schematic narrative templates is “the shining city on the hill.” But often groups disagree or hold competing historical memories. In one study, Americans were asked to list historical events “important to the foundation of America,” whether those events were positive or negative, and to list 10 historical events that “all Americans should remember.” Republicans were significantly more likely than Democrats to recall positive origin stories and less likely to remember moral atrocities such as slavery or the genocide of native Americans (Yamashiro et al., 2022).

Nations often use narrative templates as cultural schemas to make sense of contemporary public events. Narrative templates are abstract, generalized schemas that are widely shared within bounded communities. In one analysis (Wertsch, 2008), the Russian narrative template guides their interpretation of contemporary world events. Russians show large consensus on the expulsion-of-foreign-enemies narrative. According to this narrative, the Russian nation minds its own business; when powerful neighbors decide to encroach on its interests and invade, the struggle that ensues leads to an almost complete obliteration of the nation, but because of both perseverance and a sense of destiny, Russia emerges victorious. This template accommodates numerous events from Russia’s history, including the Great Patriotic War (Frederick & Coman, 2022), and is likely to serve as a frame of reference for the contemporary understanding of the country’s 2022 invasion of Ukraine. These templates, arguably, are culture specific in that different cultures develop their own idiosyncratic templates. Even though promising, this approach is still in need of empirical grounding.

Stories can also determine who does and does not get to belong. Origin stories grounded in ethnicity have the capacity to exclude large swathes of minority populations, as may be the case in parts of continental Europe (Fukuyama, 2018).^22^ Whether it is within nations or smaller organizational units, one concern that people often have is whether people such as them belong in particular spaces and groups (Walton & Cohen, 2007). When people do not feel that they belong, they often struggle to thrive—failing to live up to their potential in terms of well-being and performance. For example, minorities and first-generation college students sometimes feel that they do not belong in universities. One study (Walton & Cohen, 2011) shared stories with students that framed social adversity in school as common and temporary and encouraged them not to see difficulties as unique to them or people such as them. The stories depicted how older students had felt as though they did not belong at first, but as time went by, they felt more confident. The students were then asked to write a story to echo these experiences and to deliver it as a speech on camera. The intervention significantly raised the self-reported health, well-being, and grade point average of African American students who participated in the study.

Although stories bond group members together, they can also set them in conflict with other groups. As an example, the death of a 12-year-old Palestinian boy, Muhammad al-Durrah, was seen as one of the main events that led to the Second Intifada, a Palestinian uprising that lasted for 4 years and resulted in thousands of casualties, primarily among the Palestinians. The cause of al-Durrah’s death is widely disputed by Palestinians and Israelis, with Palestinians accusing Israeli soldiers of firing on the unarmed boy and his father. The Israeli account implies that al-Durrah’s death was caused by Palestinian fighters, who then blamed it on Israeli soldiers. This story was propagated widely both among Palestinians, providing support for a narrative of the decades of injustice and atrocities committed by Israelis, and among the Israelis, who saw this as evidence of the duplicity of Palestinians during the conflict.

In a similar vein, psychological research has documented how the same story or event could be perceived, discussed, and subsequently remembered in drastically different ways by different subcommunities (Coman et al., 2016). In a classic study (Hastorf & Cantril, 1954), Princeton and Dartmouth students who saw a football game remembered it in drastically different ways, consistent with their group allegiance. Another mechanism that could produce divergence involves the selection of different events to craft group-relevant narratives. For instance, Armenians might focus on stories that emphasize the plight of the Armenian people during the first World War, whereas their Turkish counterparts might emphasize stories that depict the Armenian population forging coalitions with the Ottoman Empire’s enemies. Antagonistic relations between different communities (Posner, 2004), the motivation to compete for scarce resources (Riek et al., 2006), and the motivation to assert group differences (Ybarra & Ramón, 2004) are factors that are likely to lead to divergence in the construction of these narratives.

In summary, societies use stories to achieve three broad goals. First, they are used to facilitate learning. Stories serve as an extension of social learning, enabling people to engage in observational learning in contexts that people rarely encounter in their day-to-day life. Stories also assist teaching by capturing pupils’ attention with engaging material. Second, stories are an effective means of persuasion. They reduce reactance and make people less likely to counterargue. They convey causal models that convince people to see things from new perspectives. They facilitate vicarious engagement with groups that people might not ordinarily engage with. Finally, stories facilitate collective action, enabling groups to address social dilemmas and coordination by establishing shared identities and common knowledge, expectations, explanations, and reputations.

## Conclusion: Stories and the Public Interest

In this article, we laid out what stories are, how they impact the mind, and how they can be leveraged in policy. We described how stories have enabled societies to transmit culture and regulate behavior over long periods of time. We discussed the features of narrative that make them so effective: engagement, identity, and meaning. Finally, we discussed three functions of stories: learning, persuading, and collective action.

Governments now regularly apply psychological theory in policy design, often testing ideas with randomized trials. Narratives have long been used in policy communication, but this work has been an art rather than a science. Here, we aimed to show that much is now known scientifically about how stories work. These principles may serve as a foundation for the integration of narratives into policy design—addressing challenges such as climate change, social cohesion, and even the economy. As with other insights from psychology, the scientific literature provides design principles for interventions that ultimately must be tested empirically. As stories of different kinds are tested more routinely, it will be possible to develop a more systematic understanding of the fit between particular story types and different contexts.

For policymakers building a narrative, we offer the following design principles:

*Start with a problem:* Research has consistently shown that the most reliable way to engage people in a narrative is to establish an inciting event and create suspense as to whether it will be resolved.
*Harness emotion:* The literature suggests that emotion is a key determinant of successful storytelling, particularly when there is flow between positively and negatively valenced events. Hence, stories are more effective when they take the audience on a journey through the ups and downs of life’s hurdles.
*Manage expectations:* Stories require a trade-off between fulfilling and violating the audience’s expectations. Without any violations, the story is entirely predictable and boring, but too many violations can lead to confusion.
*Make stories concrete:* Transportation is elicited through mental imagery. Audiences are more engaged when stories contain vivid details that enable people to feel that they can see, feel, and touch the story world.
*Leverage characters’ identities:* Characters can serve a variety of functions—whether it be to discourage negative behaviors, encourage positive ones, or shift how people think about others. Characters can also be used to signal to audiences that the communicator recognizes their perspective.
*Mind the meaning:* Leverage the causal logic of stories to convey to people what things they might value and possible ways the world works. Story content receives most cognitive processing at causal junctures.
*Context matters:* Stories come in all shapes and sizes—from complex novels such as *Ulysses* to three-line stories in newspapers. Fit the message to the task at hand.
*Treat the truth with care:* A common theme in the literature is that fictionality does not appear to limit the effect of stories on attitudes, beliefs, and behavior. This is especially important because people use schemas to organize stories and regularly fill in the gaps with stereotypes about people and other situations. To avoid spreading misinformation, governments should ground stories in available knowledge and statistics.
*Show, don’t tell:* Stories, if saturated with morals and educational content, cease to feel like entertainment, potentially subverting the policy goals. Stories that yield attitude change are effective precisely because they are less likely to elicit reactance and do not feel burdensome to consume.

## Transparency

*Editor:* Nora S. Newcombe

## Notes

1. In this article, we treat the terms “story” and “narrative” as synonyms.

2. Narratives do not necessarily have to be prototypical to have social or psychological impacts. Even basic narratives such as “he pulled himself up by his bootstraps to become CEO” or “the pound collapsed when the chancellor announced the tax cuts” can significantly affect how people organize their beliefs. For this reason, we take an inclusive definition of narrative.

3. Classic psychology research suggests that audiences are quick to interpret representations in agentic terms, even when this defies logic. In a famous experiment, Heider and Simmel (1944) showed people a short video depicting three shapes (a large triangle, a small triangle, and a small circle) moving around a screen, in and out of an opening and closing rectangular box. Most participants interpreted the movement of the shapes as if it were the purposeful behavior of animals or humans. The shapes (e.g., a big person chasing a smaller person), the shapes’ inferred emotions (e.g., fear), and the shapes’ personal characteristics (e.g., bravery, aggression) all affected participants’ interpretation of the agency of the observed visual stimuli.

4. Dennett distinguished the intentional stance from two other ways of looking at the world: the physical stance (the domain of physics and chemistry) and the design stance (the domain of engineering and biology). The physical and design stances are concerned with making sense of the world by assessing the nature or function of systems, respectively, whereas the intentional stance organizes thought through reference to agents’ beliefs and desires (Dennett, 1987).

5. The musician is Dolly Parton. Parton has appeared in 12 films and made more than 400 television appearances (YouGov America, 2022). Fame is measured by the proportion of people who have heard of or who have a popular opinion of notable people.

6. The motifs tended to fall into two broad categories: (a) adventure and tricks and (b) cosmology and etiology. Many of the motifs were virtually universal, but some were found in a handful of groups. The most common motif—found in 355 of the 958 oral traditions—was the “tasks of the in-laws” and described a narrative about how a “father or other kinsmen of hero’s wife or bride try to kill or test him and/or suggest to him difficult tasks” (Michalopoulos & Xue, 2021, p. 1998)

7. Approximately a third of the motifs depicted men as violent and aggressive and women as submissive and dependent. Women were twice as likely to be depicted in domestic roles and half as likely to be physically active.

8. The field has stirred debate within both the sciences and the humanities (Bloom, 2012; Kramnick, 2011), though this is beyond the scope of this article.

9. We know the name of the editor of the standard version of the *Epic of Gilgamesh* (1999), Sîn-lēqi-unninni, because he is listed on the text itself, and that the story is older because of the availability of fragments from earlier periods in history. The story was almost lost forever in 612 B.C.E. when the tablets were buried beneath the burning ruins of an Assyrian king’s palace, only to be rediscovered in 1853 by archaeologists near Mosul, Iraq (Damrosch, 2007).

10. Powell (1996) has argued that the Greeks’ motivation for developing their alphabet was to document these stories.

11. In these studies, the authors suggest that these correlations are causal, arguing that variation in access to media is driven by exogenous or arbitrary factors. For example, some areas received access to television earlier than others in the United States because of technical problems in the spectrum allocation, neighboring towns gained access to Fox News in different years, and the signal strength of television coming from Berlin varied across East Germany.

12. Transportation is broadly synonymous with engagement. Busselle and Bilandzic (2009) also add two features in their definition of engagement: narrative understanding (i.e., comprehension) and narrative presence (i.e., “being there”).

13. *Attention* refers to the focus of consciousness, either attending to external stimuli for the purpose of encoding information or retrieving the information from memory (Chun et al., 2011).

14. Emotions are short-lived and intense mental states characterizing evaluative, valenced reactions to stimuli (e.g., events/agents); these reactions include cognitive appraisal, arousal, subjective feeling states, and motivation (Fiske & Taylor, 2021; Ortony et al., 2022).

15. A noteworthy distinction is between liking a story and enjoying a story. It is possible to love the plot of a narrative but not enjoy the experience of reading or watching it (e.g., if the story is too descriptive or the text too small).

16. Identification is related to *parasocial interaction*, which refers to companionship between the audience and characters. Parasocial interaction operates as though it were a real-world social relationship; the audience develops lasting attachments that influence real-world aspirations (Giles, 2002). What distinguishes identification from parasocial interaction is that identification requires that people observe some characteristic that they and another person share. By contrast, people may still engage in parasocial interaction without sharing similarities, thus facilitating interaction with characters who are disliked. Hence, identification creates deep attachments but narrows the possibility for perspective taking, relative to parasocial interaction. For a general discussion of the identification construct, see Cohen (2001); Cohen et al. (2018, p. 507); de Graaf et al. (2012, pp. 805–806); and Tal-Or and Cohen (2010, pp. 403–405). Identification and transportation are related but distinct constructs (Tal-Or & Cohen, 2010). Transportation is not explicitly related to characters, and identification with characters is personal, going beyond involvement with the narrative itself. For example, in the persuasion literature (Tal-Or & Cohen, 2010), the valence of information about the hero affects the level of identification because this helps determine whether the character is likeable.

17. *Learning* refers to the encoding of novel information for long-term use. It determines the human ability to do virtually everything from walking to talking; people gain many basic capacities through learning processes (Heyes, 2018). One signature of this is humans’ unusually long developmental periods. Animals with large, complex brains have long developmental periods and have more developed capacities for socialization (Dunbar, 1993, 1998; Herculano-Houzel, 2019).

18. See Caro and Hauser (1992) for a more precise definition.

19. Scientists working at the intersection of psychology, biology, and anthropology continue to dispute the precise mechanisms underlying social learning (Heyes, 2012). Hoppitt and Laland (2013) provide a comprehensive review.

20. We do not address the use of persuasion by companies and other private interests (see van Laer et al., 2014, for a discussion).

21. Nonnarrative communication is likely to be found in opinion pieces, interviews, debates, public service announcements, music, and product advertisements.

22. One force that may mitigate this, however, is that political actors often have incentives to use stories to form broad coalitions that can attain power (R. M. Smith, 2003).
